# Hsa_circ_0030042 regulates abnormal autophagy and protects atherosclerotic plaque stability by targeting eIF4A3

**DOI:** 10.7150/thno.48389

**Published:** 2021-03-12

**Authors:** Fangpu Yu, Ya Zhang, Zunzhe Wang, Weigang Gong, Cheng Zhang

**Affiliations:** 1 The Key Laboratory of Cardiovascular Remodelling and Function Research, Chinese Ministry of Education, Chinese National Health Commission and Chinese Academy of Medical Sciences, The State and Shandong Province Joint Key Laboratory of Translational Cardiovascular Medicine, Qilu Hospital of Shandong University, 107 Wenhuaxi Road, 250012 Jinan, China.; 2 Jinan Central Hospital Affiliated to Shandong University, Jinan, Shandong Province, China.

**Keywords:** circular RNA, eIF4A3, autophagy, atherosclerosis, coronary heart disease

## Abstract

**Rationale:** Abnormal autophagic death of endothelial cells is detrimental to plaque structure as endothelial loss promotes lesional thrombosis. As emerging functional biomarkers, circular RNAs (circRNAs) are involved in various diseases, including cardiovascular. This study is aimed to determine the role of hsa_circ_0030042 in abnormal endothelial cell autophagy and plaque stability.

**Methods:** circRNA sequencing and quantitative polymerase chain reaction were performed to detect hsa_circ_0030042 expression in coronary heart disease (CHD) and human umbilical vein endothelial cells (HUVECs). Transfection of stubRFP-sensGFP-LC3 adenovirus, flow cytometry, and electron microscopy were used to identify the role of hsa_circ_0030042 in ox-LDL‒induced abnormal autophagy in vitro. Bioinformatic analysis, RNA immunoprecipitation, immunofluorescence assay and other *in vitro* experiments were performed to elucidate the mechanism underlying hsa_circ_0030042-mediated regulation of autophagy. To evaluate the role of hsa_circ_0030042 in atherosclerotic plaques and endothelial function, we measured the carotid artery tension and performed histopathology and immunohistochemistry analysis.

**Results:** hsa_circ_0030042 was significantly downregulated in CHD, while upon overexpression, it acted as an endogenous eukaryotic initiation factor 4A-III (eIF4A3) sponge to inhibit ox-LDL-induced abnormal autophagy of HUVECs and maintain plaque stability in vivo. Furthermore, hsa_circ_0030042 influenced autophagy by sponging eIF4A3 and blocking its recruitment to beclin1 and forkhead box O1 (FOXO1) mRNA, while hsa_circ_0030042-induced inhibition of beclin1 and FOXO1 was counteracted by eIF4A3 overexpression or decreased hsa_circ_0030042 binding. In high-fat-diet fed ApoE-/- mice, hsa_circ_0030042 also ameliorated plaque stability and counteracted eIF4A3-induced plaque instability.

**Conclusions:** These results demonstrate a novel pathway involving hsa_circ_0030042, eIF4A3, FOXO1, and beclin1; hence, modulating their levels may be a potential therapeutic strategy against CHD.

## Introduction

Formation and instability of atherosclerotic plaques are the pathophysiological basis for coronary heart disease (CHD) 1. Oxidised low-density lipoprotein (ox-LDL) stimulates various atherogenic pathways involving inflammatory response, foam cell formation, and cell apoptosis 2. High dose ox-LDL also triggers abnormal autophagy characterized by overexpression of beclin1, activation of autophagy, and a blockage of lysosomal degradation 3, 4. Beclin1 is involved exposing phosphatidylserine (PS) for dead-cell clearance during embryonic development, while ox-LDL induced abnormal endothelial cell autophagy 5. Forkhead box O1 (FOXO1) - a transcription factor involved in a series of intracellular functions, including autophagy, oxidative stress, mitochondrial dysfunction, and apoptosis - is involved in several atherogenic pathways in endothelial cells 6. FOXO1 inhibits autophagosome-lysosome fusion, leading to endothelial autophagic apoptosis in diabetes 7. Autophagy is a process whereby the cellular components are degraded via lipid oxidation; the subsequent death and loss of endothelial cells may result in the development of advanced plaques, increased platelet adhesion, and an accelerated thrombosis 8.

Circular RNAs (circRNAs) are a series of non-coding RNAs lacking the 5' end caps and 3' end poly (A) tails that can form closed loops 9. The ring structure allows circRNAs to resist ribonuclease R (RNase R) activity and confers a longer half-life than that of linear RNAs. Based on composition, circRNAs are classified as either exonic, intronic, or exon-intron circRNAs 10. circRNAs, especially exonic circRNAs, can serve as microRNA sponges and are responsible for the development of various diseases, including cancer, myocardial infarction, atherosclerosis, and neurodegenerative diseases 11. HRCR, a heart-related circRNA, may protect against pathological hypertrophy and heart failure by targeting miR-223 12. Conversely, circRNAs may also function as protein sponges. Extensive binding of circPABPN1 to HuR prevents HuR binding to PABPN1 mRNA and lowers its translation 13. circACR represses cardiomyocyte autophagy through activation of Pink1 expression by directly binding to Dnmt3B and blocking Dnmt3B-mediated methylation of the Pink1 promoter 14. However, it remains unclear how autophagy is regulated by circRNAs in the context of atherosclerotic plaque stability.

Eukaryotic initiation factor 4A-III (eIF4A3) - a core protein of the exon junction complex (EJC) - is involved in RNA splicing, nonsense-mediated decay, translation, and localisation 15. In EJC splicing, spliced mRNAs exhibit higher stability and translation 16. EJC can also enhance translation directly through interactions of eIF4A3 with SKAR, thereby facilitating mTOR signalling 17. As an RNA-binding protein (RBP), eIF4A3 binds to canonical mRNA sites, 20-24 nucleotides upstream of exon-exon junctions, or to non-canonical sites spread across the mRNA via its DEAD-box domain 18; this binding may be affected by various proteins. EJC influences events primarily in the nucleus, while eIF4A3 binds to several spliced mRNAs, suggesting that EJC deposition is a regulated event 19-21. Moreover, lncRNA-H19 binds to eIF4A3 and inhibits its recruitment to cyclin D1, cyclin E1, and CDK4 22. However, whether eIF4A3 is sponged by circRNA or is involved in plaque stability regulation remains unknown.

Our current study reveals that hsa_circ_0030042 - a CHD related circRNA - inhibits the expression of FOXO1 and beclin1, which are involved in abnormal autophagy, by recruiting eIF4A3 into HUVECs and that hsa_circ_0030042 increases plaque stability and counteracts eIF4A3-induced plaque instability in ApoE^-/-^ mice.

## Methods

Expanded materials and methods are available in the Supplementary Materials.

### Ethics statement

The study was approved by a local committee on the use of human samples for experimental studies at Qilu Hospital of Shandong University, Shandong, China (Prot. KYLL-2019-080). Informed consent was given prior to the inclusion of subjects in the study. Human coronary arteries were acquired from six brain-dead (DBD) organ donors. Written informed consents were provided by the donor families after donor brain injury and prior to enrolment. The consent included generation of open-access genetic sequencing data and publication in open access journals in line with Wellcome Trust policy. All experimental methods were conducted in accordance with the Helsinki Declaration and the ARRIVE Guidelines on the protection of animals used for scientific purposes.

### Generation of stable hsa_circ_0030042-overexpressing HUVECs

To achieve lentivirus-mediated hsa_circ_0030042 overexpression, the lentivirus vector pLO-ciR and pLO-ciR harboring hsa_circ_0030042 (pLCDH-c0030042) were purchased from Geneseed (Guangzhou, China) and the cycle junction site was validated by sequencing after PCR (forward primer, 5'-TGGATGGAGATACATTGGATT-3'; reverse primer, 5'-ATTGAGCATCCACCAAGAAC-3'). Then, the pLO-ciR and pLCDH-c0030042 were used to independently transfect HUVECs at an MOI 10. HUVECs stably expressing hsa_circ_0030042 (c0030042) and pLO-ciR HUVECs (circ-N.C) were obtained by puromycin 1 μg/ml selection. qPCR was used to test the expression of hsa_circ_0030042.

To achieve lentivirus-mediated human eIF4A3 overexpression, lentiviral HBLV-h-eIF4A3-GFP-PURO and HBLV-GFP-PURO were purchased from Hanbio (Shanghai, China). We then used HBLV-h-eIF4A3-GFP-PURO and HBLV-GFP-PURO to independently transfect HUVECs at MOI 10. HUVECs stably expressing eIF4A3 (h-eIF4A3-GFP) and GFP alone (GFP-N.C) were obtained by puromycin 1 µg/mL selection. Western blot was used to evaluate the expression of eIF4A3.

### Transfection of circ-delete plasmid

Using the catRAPID and Vienna RNA algorithm for circRNA secondary structure and RNA-protein interaction, hsa_circ_0030042-eIF4A3 binding was predicted to be mediated through three major RNA regions (180‒227, 812‒867, and 1082‒1137 nucleotides). With the plasmid used in pLCDH-c0030042, we constructed special hsa_circ_0030042 overexpression plasmid in which three main binding sites of hsa_circ_0030042 to eIF4A3 were deleted (circ-delete). The cycle junction was the same as pLCDH-c0030042. The circ-delete plasmids were transfected in 293A cells or HUVECs using Lipofectamine 3000.

### siRNA and RNA interference

Upon reaching 40-50% confluence, HUVECs were transfected with specific siRNA (GenePharma, Shanghai, China and Geneseed, Guangzhou, China) or negative control siRNA (GenePharma, Shanghai, China) using Lipofectamine 3000 (Thermo Fisher Scientific, Waltham, MA, USA) in Opti-MEM (Gibco, Thermo Fisher Scientific, Waltham, MA, USA); hsa_circ_0030042 siRNA target sequence, 5'-GCAGAATTCAATTCGTCAT-3'; eIF4A3 siRNA target sequence, 5'-GCAATCCAGCAACGAGCAATT-3'. After 6 h transfection, the medium was replaced with complete ECM, and the cells were cultured for an additional 24-48 h.

### Fluorescence *in situ* hybridization (FISH)

To observe hsa_circ_0030042 in HUVECs and aortic tissue, we performed FISH using a Fluorescent *In situ* Hybridization Kit (Ribo, Guangzhou, China). In brief, samples were fixed and pretreated with 4% paraformaldehyde and PBS with 0.5% Triton X-100. Then, h-circ0030042 Probe Mix and h-18s FISH Probe Mix (Ribo) were used separately to hybridize with the samples at 37 °C, overnight. Finally, after DAPI staining of the nuclei, the slides were imaged using confocal laser scanning microscopy.

### RNA Immunoprecipitation (RIP)

RNA immunoprecipitation was performed using eIF4A3-specific antibody (ab180573), Ago2-specific (ab186733), and HuR-specific (lot.2880766 Millipore). IgG antibody was used as negative control. The immunoprecipitation was conducted using a Magna RIP kit (Millipore) according to manufacturer's instructions. In brief, HUVECs were lysed in lysis buffer containing proteinase inhibitor cocktail and RNase inhibitor. The lysate was incubated overnight with antibody-coupled magnetic beads on rotation at 4 °C. Bead-antibody complexes were subsequently washed thoroughly with RIP wash buffer. RNA purification and extraction used proteinase K digestion and phenol chloroform. With salt solution I, salt solution II and precipitate enhancer in ethanol, -80 °C for overnight, RNAs were obtained for the downstream RNA detection.

### Atherosclerosis animal model construction

A total of 100 eight-week-old male ApoE^-/-^ mice were purchased from the Laboratory Animal Resources, Chinese Academy of Sciences (Beijing), fed a western diet (i.e., a high-fat diet with 40% fat and 1.25% cholesterol) for 10 weeks, and randomly divided into four groups (n = 25 each): An empty lentivirus group (GFP-N.C), a hsa_circ_0030042 lentivirus group (c0030042), a human eIF4A3 lentivirus group (h-eIF4A3-GFP), and a hsa_circ_0030042 and human eIF4A3 lentivirus co-transfection group (c0030042+h-eIF4A3). A 200 μL suspension (2×10^8^ TU/mL empty lentivirus or hsa_circ_0030042 lentivirus or human eIF4A3 lentivirus or mixed) was injected into each mouse through the tail vein. After 6 weeks of atherogenic food feeding, mice were subjected to artery plaque measurement, including size, components, and vulnerability. Isoflurane (4%) mixed with oxygen (100%, 0.5-1.5 L/min) was used as inhalable anaesthesia prior to harvesting the heart and aorta. Mice were fully anaesthetized, and following perfusion with 0.9% saline, hearts with aortae were harvested; mice were then euthanized by cervical dislocation after the harvest was completed.

### Histopathology and immunohistochemistry

The mice aortic tissues were dissected, removed, fixed in 4% formaldehyde overnight at 4 °C. The aortic tissues were embedded in OCT compound and sliced into 5-μm-thick sections. The frozen sections were stained with hematoxylin and eosin to observe plaque morphology, while oil red O and picrosirius red stainings were used to observe lipids and collagen, respectively. After blocking in 5% bovine serum albumin (BSA) in PBS, the sections were incubated with primary antibodies overnight at 4 °C and then with an HRP Detection System (ZSGB-BIO, Beijing, China). Detection was subsequently conducted using DAB (ZSGB-BIO). The areas of collagen, VSMCs, lipids, and macrophages were recorded as the percentage of positive area to that of the plaque using a 20 high-power fields (20×). The images were quantified using Image-Pro Plus 6.0 software (Media Cybernetics, Rockville, MD, USA). The vulnerability index of the plaque was calculated using the following formula: (macrophage staining% + lipid staining%) / (VSMC staining% + collagen staining%). The antibodies used for immunohistochemistry were: Anti-αSMA antibody (1:1,000, ab5694), anti-MOMA2 antibody (1:50, ab33451), anti-eIF4A3 antibody (1:1,000, ab180573), anti-FOXO1 antibody (1:100, 2880, CST), anti-beclin1 antibody (1:500, ab62557) and anti-LC3B antibody (1:200, ab48394).

### Measurement of carotid artery tension in mice

A 2mm carotid artery tissues without plaques were immersed in PSS buffer (130 mM NaCl, 4.7 mM KCl, 1.18 mM KH2PO4, 1.17 mM MgSO4·7H2O, 24.9 mM NaHCO3, 5.5 mM glucose, 0.026 mM EDTA and 1.6 mM CaCl2) and suspended by two tungsten wires mounted to a vessel myograph system (Danish Myo Technology A/S, USA). After undergoing an equilibration period, rings were treated with phenylephrine (NE) to induce contraction. Then, acetylcholine (Ach, 10-9-10-7 M) was injected at the plateau of the NE-induced contraction.

### Statistical analysis

Data are presented as mean ± SD, medians (quartiles), or proportions when appropriate. Continuous data are presented in scatter/dot plots together with the average/error bars. At least four independent experiments for each experimental and animal group were performed. Two-group comparisons were analysed using Student's t-test. One-way or two-way ANOVA were used for multiple comparisons in SPSS 25.0 (SPSS Inc., Chicago, IL, USA) or GraphPad Prism 8 (GraphPad Software, CA, USA). The assumption of normality was tested by the Shapiro-Wilks test. All statistical tests were two-tailed and P < 0.05 was considered statistically significant.

## Results

### hsa_circ_0030042 expression is downregulated in CHD

**Figure 1:**
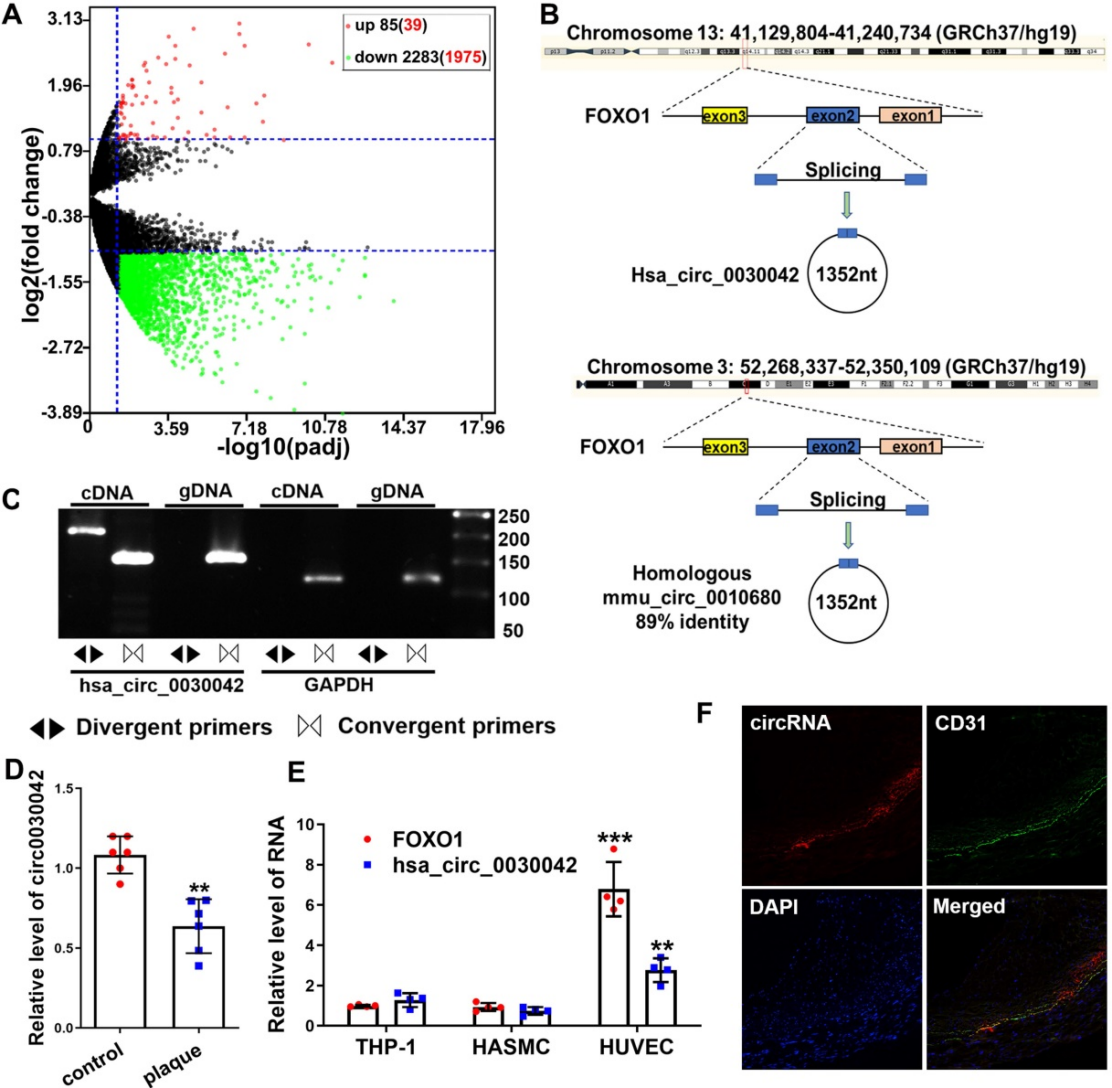
circRNA sequencing uncovered CHD-related hsa_circ_0030042, which was validated using qRT-PCR in human atherosclerotic plaques. **A.** Volcano plot of sequenced PBMC circRNAs. X-axis: padj expressed as -log10; Y-axis: Fold change expressed as log2. The vertical line represents a padj of 0.05. The number in brackets indicated circRNAs having annotations in circRNAs. Compared with the control, the red points represent circRNAs with a fold change ≥ 2 and padj<0.05, and the green points represent circRNAs with a fold change ≤ 0.5 and padj<0.05. The horizontal line represents a padj of 0.05. Data were performed in DEseq2 R package and the padj were the p-value adjusted using the Benjamini-Hochberg procedure to control the false discovery rate (FDR). **B**. An illustration of the production of hsa_circ_0030042 and its homologous circRNA mmu_circ_0010680 in the mouse. **C**. Specific divergent primers amplified hsa_circ_0030042 in cDNA but not genomic DNA (gDNA) in HUVECs. GAPDH was used as a linear control. **D**. qRT-PCR validated hsa_circ_0030042 in human coronary arteries with plaques or matched controls without plaques. Data are presented as mean ± SD. Student's t-test. **p < 0.01. n = 6 pairs. E. FOXO1 mRNA and hsa_circ_0030042 levels in different vascular cells. Data are presented as mean ± SD. Student's t-test. Compared with THP-1, **p < 0.01, ***p < 0.001. n = 4. F. The location of hsa_circ_0030042 and endothelial cells were co-detected with FISH.

We sequenced 70 CHD and 30 control RNA samples from peripheral blood mononuclear cells (PBMCs) to investigate the circRNA profile in CHD and found that 85 and 2,283 (including hsa_circ_0030042) circRNAs were significantly up- and downregulated compared with those in controls (Figure 1A). Of the top 50 downregulated and 50 upregulated circRNAs, hsa_circ_0030042 (with log2 (fold change) of -3.17 and padj of 1.84×10^-6^) was identified as the circRNA of interest. Its parental gene FOXO1 is closely associated with CHD, and was the only exonic circRNA with a length of <2,000 nt (Table S1). hsa_circ_0030042 was sequenced in HUVECs with high conservation according to circBase (Figure 1B). To confirm the presence of hsa_circ_0030042 in HUVECs, we designed specific hsa_circ_0030042 divergent primers and detected an abundance of hsa_circ_0030042 (Figure 1C). Then, we detected hsa_circ_0030042 in six matched human coronary samples using qRT-PCR. Noticeably, in comparison with that in coronaries without plaque, hsa_circ_0030042 was downregulated in coronaries with plaques (Figure 1D). The dysregulation of hsa_circ_0030042 in human coronary artery plaque was in line with the results of the subsequent sequencing of blood samples, demonstrating its potential as a CHD biomarker. We also measured the homologous mmu_circ_0010680 in 3-month-old C57BL/6 and ApoE^-/-^ mice using qRT-PCR, and found that compared with C57BL/6, mmu_circ_0010680 was downregulated in the aorta and liver and upregulated in the heart and kidney of ApoE^-/-^ mice (Figure S1A). Furthermore, qRT-PCR showed that hsa_circ_0030042 and FOXO1 were much higher in HUVECs than Human Aortic Smooth Muscle Cells (HASMCs) and THP-1 cells (Figure 1E), while results from FISH indicated that, in human coronary artery, hsa_circ_0030042 were primarily located in the endothelial cells (Figure 1F). Therefore, we selected HUVECs to further study hsa_circ_0030042 in CHD.

### eIF4A3 is sponged by hsa_circ_0030042 in HUVECs

**Figure 2:**
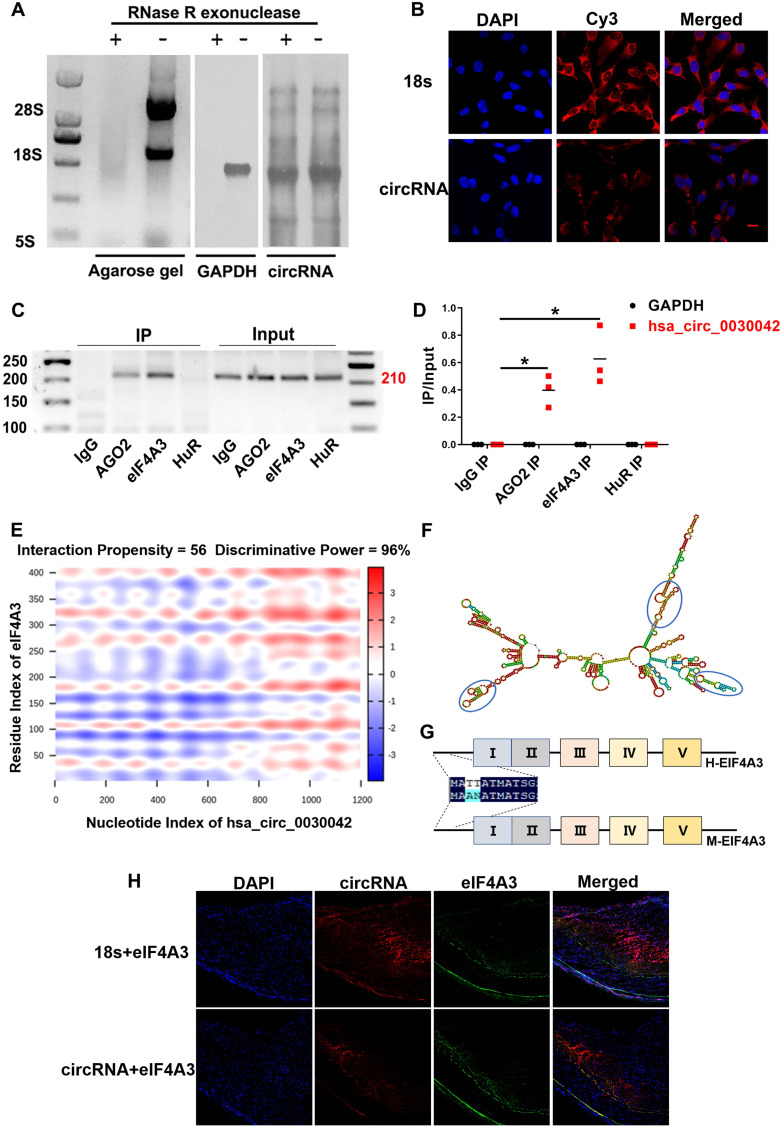
hsa_circ_0030042 localised in the cytoplasm and interact with eIF4A3. **A.** Northern blot detected circular but not linear CHD-related hsa_circ_0030042 in HUVEC. Agarose gel, HUVEC total RNA agarose gel electrophoresis; GAPDH, GAPDH probe; circRNA, a specific probe of hsa_circ_0030042. **B.** Fluorescence *in situ* hybridisation assay (FISH) showing that hsa_circ_0030042 is localised in the cytoplasm of HUVECs. 18s rRNA was used as control. hsa_circ_0030042 and 18s rRNA probes were all labelled with Cy3. Scale bar, 20 µm. **C & D.** RNA immunoprecipitation of AGO2, eIF4A3, and HuR from HUVECs. hsa_circ_0030042 and GAPDH qRT-PCR products were determined using agarose gel electrophoresis in (**C**), and the relative immunoprecipitated (IP) / Input ratios are plotted in (**D**). Data are presented as mean ± SD. Student's t-test. ***p < 0.001. n = 4. **E.** Prediction of RNA-protein interaction of hsa_circ_0030042 with eIF4A3 using the catRAPID algorithm.** F.** Secondary structure prediction of hsa_circ_0030042 using the Vienna RNA Web Services. Predicted top three binding sites of hsa_circ_0030042 with eIF4A3 were shown in the blue circle. **G.** The scheme illustrating the high homology of eIF4A3 in humans and mice. Five predicted binding sites with hsa_circ_0030042 are shown in Roman numerals. **H.** FISH analysis showed that hsa_circ_0030042 was co-localised with eIF4A3 in the human coronary artery. 18s rRNA was used as control. Scale bar, 100 µm.

hsa_circ_0030042 circularisation was assayed using northern blot in HUVECs (Figure 2A). FISH analysis showed that hsa_circ_0030042 in HUVECs was mostly located in the cytoplasm, which acts as a miRNA or protein sponge (Figure 2B). To determine the potential function of hsa_circ_0030042, we used RPISeq (version 1.0) to identify possible interaction with Argonaute 2 (AGO2), eIF4A3, and Human antigen R (HuR) (Table S2), which were selected for RNA binding protein immunoprecipitation (RIP). Agarose gel electrophoresis results showed that with the anti-AGO2 and anti-eIF4A3 antibody immunoprecipitation, endogenous hsa_circ_0030042 was specifically enriched, while anti-HuR complex could not bind to this circRNA (Figure 2C-D). RIP indicated that hsa_circ_0030042 might function as a miRNA and eIF4A3 sponge. Using the catRAPID and Vienna RNA algorithm for circRNA secondary structure and RNA-protein interaction, hsa_circ_0030042-eIF4A3 binding was shown to be mediated through three major RNA regions (180‒227, 812‒867, and 1082‒1137 nucleotides), with RNA stem-loop structures (Figure 2E-F), and through the five domains (61‒112, 111‒162, 176‒227, 251‒302, and 336‒387 aa) of eIF4A3 protein (Figure 2G), respectively. These five domains in eIF4A3 are highly homologous in humans and mice. FISH analysis showed that hsa_circ_0030042 was co-localised with eIF4A3 in the human coronary artery (Figure 2H).

### hsa_circ_0030042 inhibits ox-LDL induced abnormal autophagy in HUVECs

**Figure 3:**
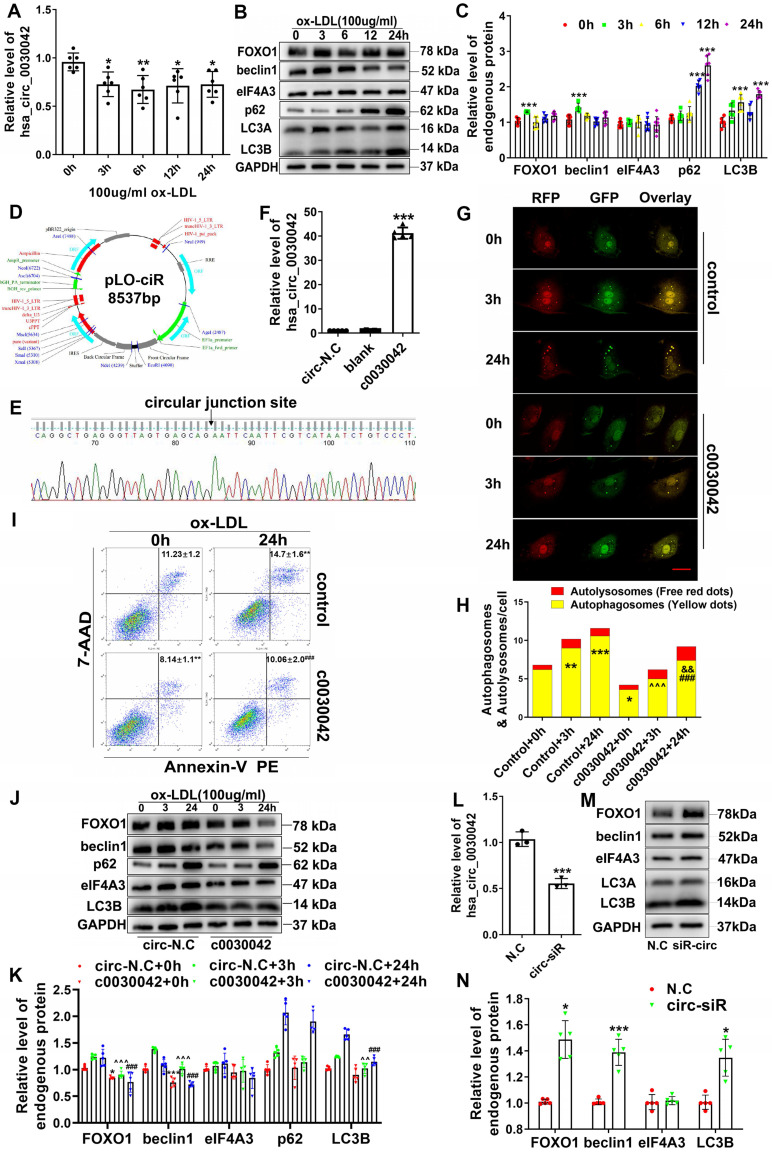
hsa_circ_0030042 inhibits ox-LDL induced abnormal autophagy in HUVECs. **A.** Relative level of hsa_circ_0030042 in ox-LDL treated HUVECs. X-axis: Stimulation time for 100 µg/mL ox-LDL. One-way ANOVA. *p < 0.05, **p < 0.01. n = 6. **B & C.** Relative protein level of FOXO1, beclin1, eIF4A3, and LC3B in ox-LDL treated HUVECs. One-way ANOVA. Compared with 0 h, ***p < 0.001. n ≥ 6. **D - F.** Overexpression of hsa_circ_0030042 in HUVECs. **D**. The hsa_circ_0030042 lentivirus vector pLO-ciR. **E**. the circular junction site is shown in. **F.** Relative hsa_circ_0030042 level of stable hsa_circ_0030042 overexpression HUVECs (c0030042) to that in empty vector transfected HUVEC (circ-N.C). One-way ANOVA. ***p < 0.001. n = 5. **G & H.** With stubRFP-sensGFP-LC3 adenovirus transfection, ox-LDL was used to treat c0030042 and HUVEC under various time points (control). Confocal microscopy showed autophagy flux in (**G**). Yellow dots: autophagosomes; Free red dots: autolysosomes. Scale bar, 20 µm. **H**. result is quantification (**H**). Two-way ANOVA. Compared with control + 0 h, *p < 0.05, **p < 0.01, ***p < 0.001; compared with c0030042+0h, ###p < 0.001; compared with control+3 h, ^^^p < 0.001; compared with control + 24 h, &&p < 0.01. n = 5. **I.** Flow cytometric analysis for quantification of early apoptotic cells in ox-LDL-treated c0030042 and HUVEC (control). Percentages of Annexin V-PE positive cells (annexin V+/7-AAD- and annexin V+/7-AAD+) are indicated. One-way ANOVA. Compared with control + 0h, **p < 0.01; compared with control+24h, ###p < 0.001. n = 6.** J & K.** Western blot for quantifying protein levels in ox-LDL-stimulated c0030042 and circ-N.C under various time points. Two-way ANOVA. Compared with circ-N.C + 0 h, *p < 0.05, ***p < 0.001; compared with circ-N.C + 3 h, ^^p < 0.01, ^^^p < 0.001; compared with circ-N.C + 24 h, ###p < 0.001. n = 5. **L - N.** HUVECs were transiently transfected with negative control (N.C) and hsa_circ_0030042 siRNA (circ-siR) for 48 h.** L**. Relative level of hsa_circ_0030042. **M & N.** Western quantification of protein levels. Student's t-test. Compared with N.C, *p < 0.05, ***p < 0.001. n = 5. Data are presented as mean ± SD.

Since ox-LDL induces abnormal autophagy at a high-dose, we applied it to HUVECs and used qRT-PCR to determine hsa_circ_0030042 level (Figure 3A); protein levels of FOXO1, beclin1, eIF4A3, and LC3B were also detected (Figure 3B). hsa_circ_0030042 level was reduced, accompanied by elevated FOXO1, beclin1, and LC3B, by 100 µg/ml ox-LDL, with no quantitative change in eIF4A3 level (Figure 3C). To determine whether hsa_circ_0030042 participates in the ox-LDL-induced abnormal autophagy pathway, we constructed hsa_circ_0030042 stable overexpression HUVECs (c0030042) and GFP-control HUVECs (circ-N.C) (Figure 3D-F). After transfection with stubRFP-sensGFP-LC3 adenoviruses for 72 h, cells were exposed to 100 µg/mL ox-LDL for 0, 3, and 24 h. Confocal microscopy showed that ox-LDL upregulated autophagosome puncta (yellow dots), with rare autolysosomes (red dots) over time. The autophagosome puncta of c0030042 group were lower than those of HUVEC (control) group (Figure 3G-H). Protein levels of endogenous FOXO1, beclin1, and LC3B in ox-LDL-induced c0030042 were lower than those in the circ-N.C group (Figure 3J-K). Flow cytometry results showed that hsa_circ_0030042 overexpression decreased proportion of Annexin V-positive cells after treatment with ox-LDL for 24 h (Figure 3I). Endogenous levels of FOXO1, beclin1, and LC3B in hsa_circ_0030042 siRNA-transfected HUVECs (circ-siR) were higher than those of the negative control (N.C). eIF4A3 showed no quantitative change (Figure 3L-N). To determine whether hsa_circ_0030042 regulate autophagy under the atherosclerotic plaque environment, we exposed human coronary artery endothelial cells (HCAECs) to unidirectional pulsatile shear stress (PSS) (12 dyne/cm^2^, PSS) or oscillatory shear stress (OSS) (0 ± 4 dyne/cm^2^, OSS) for 12h. Compared with the static condition, OSS enhanced autophagy (expression of LC3B) by 6-8 folds, while PSS caused a gradual decline. hsa_circ_0030042 reduced FOXO1 and beclin1 and alleviate autophagy under the haemodynamic conditions (Figure S2). These indicate that ox-LDL could stimulate abnormal autophagy by upregulating beclin1 and FOXO1 and accumulating excessive autophagosome puncta with obstructed metabolic ability, which may lead to apoptosis or death of endothelial cells, while hsa_circ_0030042 could mitigate the ox-LDL-induced accumulation of autophagosomes and reduce PS exposure.

### hsa_circ_0030042 inhibits abnormal autophagy through obstructing the recruitment of eIF4A3 to beclin1 and FOXO1 mRNA

**Figure 4:**
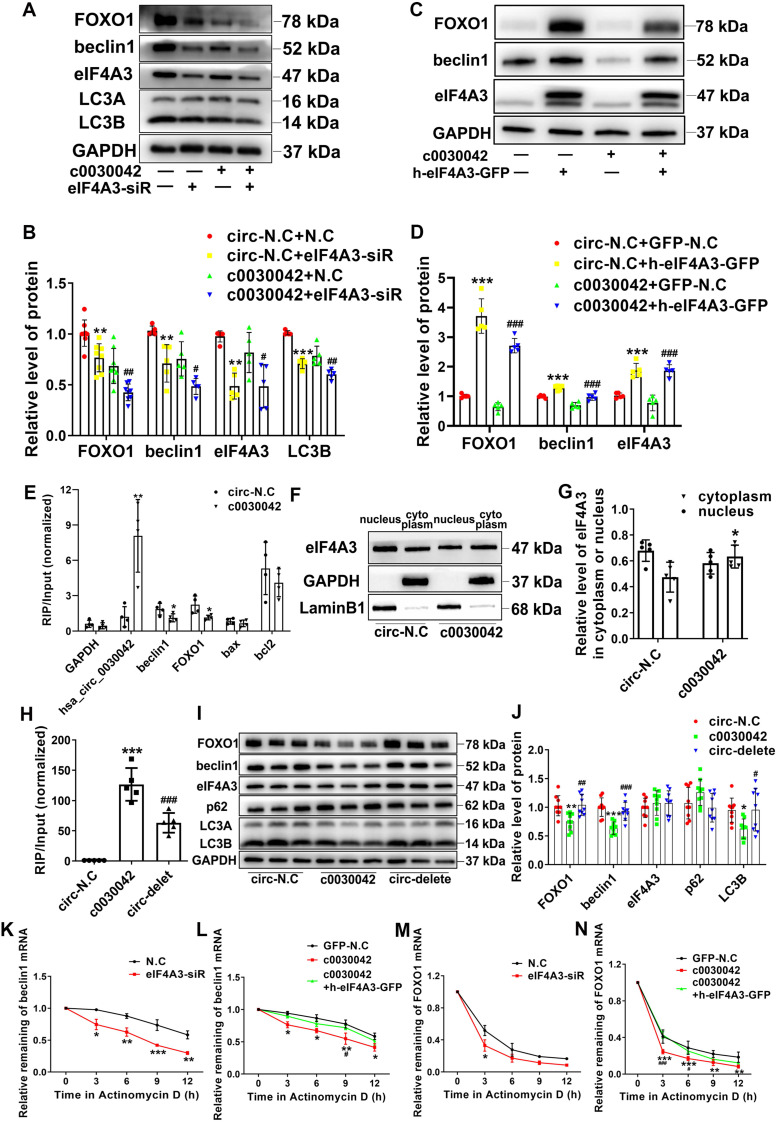
hsa_circ_0030042 inhibits excessive autophagy through obstructing the recruitment of eIF4A3 to beclin1 and FOXO1 mRNA. **A & B.** Stable hsa_circ_0030042 overexpression HUVECs (c0030042) and empty vector transfected HUVECs (circ-N.C) were transiently transfected with negative control (N.C) and eIF4A3 siRNA for 48 h. FOXO1, beclin1, eIF4A3, and LC3B protein were quantified using western blot. One-way ANOVA, compared with circ-N.C+N.C, **p < 0.01, ***p < 0.001; compared with c0030042+N.C, #p < 0.05, ##p < 0.01. n ≥ 5. **C & D.** eIF4A3 lentivirus (h-eIF4A3-GFP) and empty GFP lentivirus (GFP-N.C) were transfected into c0030042 and circ-N.C, respectively. FOXO1, beclin1 and eIF4A3 were detected using western blot. Compared with circ-N.C+GFP-N.C, ***p < 0.001; compared with c0030042+GFP-N.C, ###p < 0.001. n = 5. **E.** RNA immunoprecipitation (RIP) of eIF4A3 from c0030042 and circ-N.C group. hsa_circ_0030042, beclin1, FOXO1, Bax, Bcl2, and GAPDH mRNA bound to eIF4A3 were determined using qRT-PCR. Input of c0030042 was normalised to that of the circ-N.C group. Student's t-test. *p < 0.01, **p < 0.01. n = 4. **F & G.** Western blot showing relative level of eIF4A3 in the cytoplasm or nucleus in the c0030042 and circ-N.C groups (**F**), and the ratio was quantified (**G**). Student's t-test. *p < 0.05. n = 5. **H.** RIP of eIF4A3 from circ-N.C, c0030042, and circ-delete groups. The eIF4A3-bound hsa_circ_0030042 hsa_circ_0030042 was determined using qRT-PCR. Input of c0030042 and circ-delete was normalised to that of the circ-N.C group. One-way ANOVA, compared with circ-N.C, ***p < 0.001; compared with c0030042, ###p < 0.001. n = 5.** I & J.** Western blot showing the relative level of FOXO1, beclin1, eIF4A3, p62 and LC3B. One-way ANOVA. Compared with circ-N.C, *p < 0.05, **p < 0.01, ***p < 0.001; compared with c0030042, #p < 0.05, ##p < 0.01, ###p < 0.001. n = 9. **K - N.** After Actinomycin D treatment, the mRNA stability of beclin1 and FOXO1 in indicated cells was determined using qRT-PCR. Two-way ANOVA. Compared with N.C, *p < 0.05, **p < 0.01, ***p < 0.001; compared with c0030042+h-eIF4A3-GFP, #p < 0.05, ###p < 0.001. n = 4. Data are presented as mean ± SD.

To confirm the role of eIF4A3 in hsa_circ_0030042 regulation of the autophagy pathway, c0030042 and circ-N.C groups were transfected with eIF4A3 siRNA and negative control siRNA (N.C), respectively. With eIF4A3 depression, FOXO1, beclin1, and LC3B were significantly decreased in both the c0030042 and circ-N.C groups (Figure 4A-B). Compared with circ-N.C, c0030042 group showed decreased formation of autophagosomes induced by ox-LDL and eIF4A3 depression could further decrease the autophagic vacuoles in both c0030042 and circ-N.C group (Figure S3A-B). The c0030042 group transfected with eIF4A3 overexpression lentivirus (c0030042+h-eIF4A3-GFP) showed increased FOXO1 and beclin1 expression compared with the c0030042 group transfected with empty GFP lentivirus (c0030042+GFP-N.C), similar to the trend observed in the circ-N.C group (Figure 4C-D). The results demonstrated that eIF4A3 participated in hsa_circ_0030042 regulation of FOXO1 and beclin1. Furthermore, using RIP on c0030042 and circ-N.C groups, we found that eIF4A3 and hsa_circ_0030042 were upregulated in c0030042. Meanwhile, the enriched FOXO1 and beclin1 mRNA on eIF4A3 in the c0030042 group was much less than that in the circ-N.C group, while apoptosis-related Bax and Bcl2 mRNA showed no significant change (Figure 4E). Moreover, when nuclear and cytoplasmic proteins were separately extracted, total eIF4A3 remained unchanged, while eIF4A3 ratio of the cytoplasm to that of the nucleus in the c0030042 group was higher than that in the circ-N.C group (Figure 4F-G). Immunofluorescence of circ-N.C and c0030042 group showed increased eIF4A3 cytoplasmic localization in c0030042 group. (Figure S3C). To verify that FOXO1 and beclin1 suppression was caused by hsa_circ_0030042 competitive co-binding with eIF4A3, we constructed three-binding-site deletion hsa_circ_0030042 overexpression plasmids (circ-delete) and transfected them into 293A cells. RIP with eIF4A3 antibody showed that eIF4A3-bound hsa_circ_0030042 was decreased in circ-delete compared with that in the normal hsa_circ_0030042 overexpression group (c0030042) (Figure 4H). In HUVECs, the circ-delete group showed no significant change in FOXO1, beclin1, and LC3B protein expression levels compared with those in the empty vector-transfected group (circ-N.C) (Figure 4I-J). Since eIF4A3 activity is reflected by its target mRNA 23, to determine hsa_circ_0030042 effects on eIF4A3, we used Actinomycin D to block transcription for different time points while the remaining beclin1 and FOXO1 mRNA was assessed using qRT-PCR. As expected, eIF4A3-siRNA decreased mRNA stability of beclin1 and FOXO1 (Figure 4K, 4M); in addition, hsa_circ_0030042 also decreased beclin1 and FOXO1 mRNA stability, while the rebound of beclin1 and FOXO1 mRNA stability occurred with upregulation of eIF4A3 (Figure 4L, 4N). This indicates that hsa_circ_0030042 inhibits FOXO1 and beclin1 expression by affecting eIF4A3 nuclear translocation and obstructing eIF4A3 recruitment on their mRNAs.

### hsa_circ_0030042 regulates autophagy in vivo

**Figure 5:**
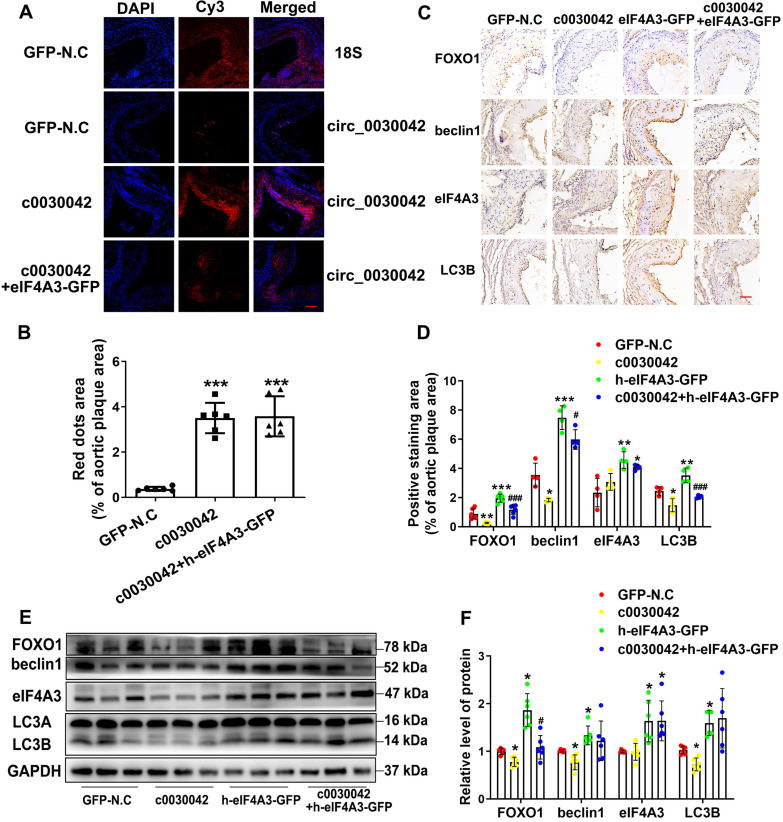
hsa_circ_0030042 regulates autophagy *in vivo* through eIF4A3. Eight-week male ApoE^-/-^ mice were fed high-fat diet for 10 weeks and randomly divided into lentivirus GFP-N.C, c0030042, h-eIF4A3-GFP, and c0030042+h-eIF4A3-GFP groups. With transfection for another 6 weeks with atherogenic food, mice were processed for aortic plaques. **A & B.** FISH showed overexpression of hsa_circ_0030042 in the aortic root plaques four weeks after lentivirus transfection; 18s rRNA was used as control. hsa_circ_0030042 and 18s rRNA probes were all labelled with Cy3. Scale bar, 50µm. Data are presented as mean ± SD. One-way ANOVA, compared with GFP-N.C, ***p < 0.001. n = 6. **C & D.** Representative immunohistochemical stained images and quantification of FOXO1, beclin1, eIF4A3 and LC3B levels in aortic root plaques. Data are presented as mean ± SD. One-way ANOVA. Compared with GFP-N.C, *p < 0.05, **p < 0.01, ***p < 0.001; compared with h-eIF4A3-GFP, #p < 0.05, ###p < 0.001. n ≥ 4.** E & F.** Western blot of FOXO1, beclin1, eIF4A3, and LC3B in aorta of the four groups of transfected mice. Scale bar, 50 µm. Data are presented as mean ± SD. One-way ANOVA. Compared with GFP-N.C, *p < 0.05; compared with h-eIF4A3-GFP, #p < 0.05. n ≥ 4.

In mice, FISH of hsa_circ_0030042 was used to ensure that hsa_circ_0030042 overexpression in aortic plaques was effective (Figure 5A-B). We found no difference in mmu_circ_0010680 level between the c0030042 and the GFP-N.C groups *in vivo* (Figure S1B) which indicate that modulation of hsa_circ_0030042 may have no effects on mmu_circ_0010680 expression. However, with RIP of eIF4A3 in c0030042 group, we found that hsa_circ_0030042 could also sponge murine eIF4A3 in Figure S4. The result showed that eIF4A3 could sponged by mmu_circ_0010680 and hsa_circ_0030042 *in vivo*. Immunohistochemistry showed that enforced hsa_circ_0030042 expression (c0030042) in mice decreased FOXO1, beclin1, and LC3B. Human eIF4A3 (h-eIF4A3-GFP) increased FOXO1, beclin1, and LC3B, while hsa_circ_0030042 overexpression (c0030042+ h-eIF4A3) antagonised this function (Figure 5C-D). Western blot results were in line with those of immunohistochemistry (Figure 5E-F). These results establish that hsa_circ_0030042 regulates autophagy at the plaque level.

### hsa_circ_0030042 increases advanced atherosclerotic plaque stability *in vivo*

**Figure 6:**
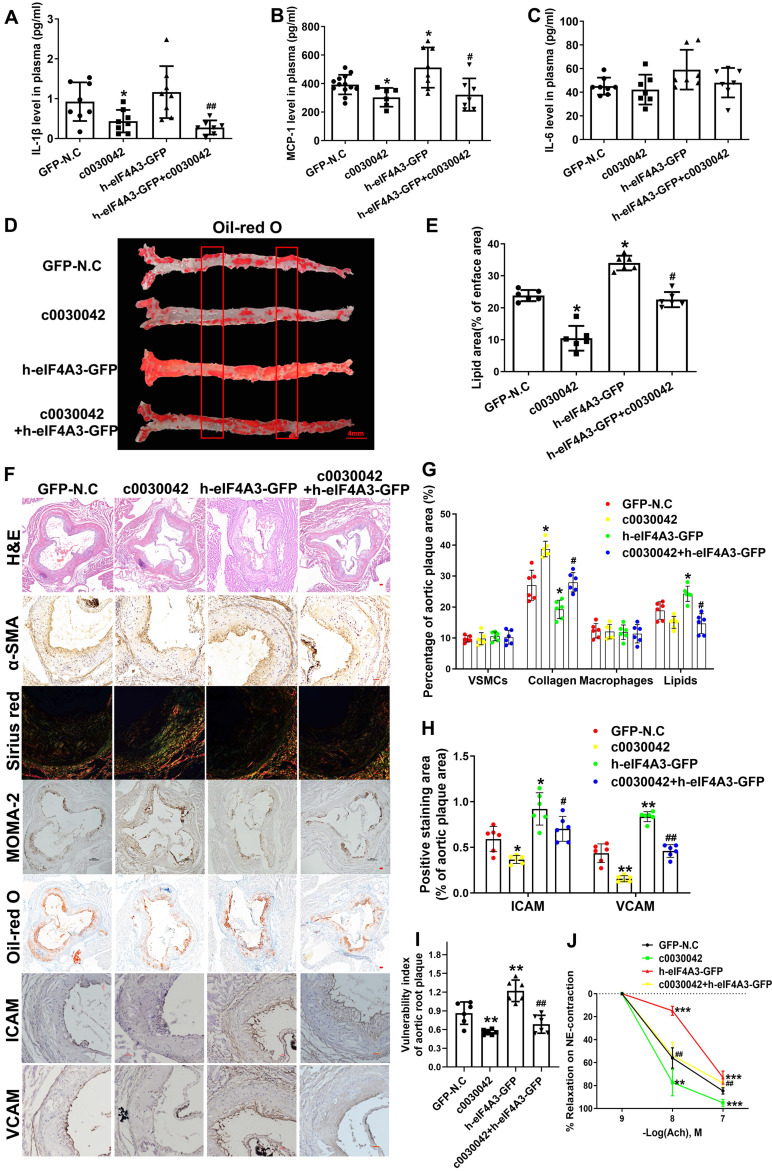
hsa_circ_0030042 effects atherosclerotic plaque stability *in vivo*. **A - C.** Enzyme-linked immunosorbent assay (ELISA) of plasma level of IL-1β (**A**), MCP-1 (**B**), and IL-6 (**C**). Data are presented as mean ± SD. One-way ANOVA, compared with GFP-N.C, *p < 0.05; compared with h-eIF4A3-GFP, #p < 0.05, ##p < 0.01. n ≥ 6. **D.** The position of the thoracic and abdominal aortae sliced section. **D & E.** En face Oil Red O staining of aortas and quantification in four groups of mice (GFP-N.C, c0030042, h-eIF4A3-GFP and c0030042+h-eIF4A3-GFP group). Data are presented as mean ± SD. One-way ANOVA, compared with GFP-N.C, *p < 0.05, **p < 0.01; compared with h-eIF4A3-GFP, #p < 0.05. n = 6. **F - H.** Representative immunohistochemical staining and quantification of plaque contents in aortic root plaques. Scale bar, 50µm. Data are presented as mean ± SD. One-way ANOVA, compared with GFP-N.C, *p < 0.05, **p < 0.01, ***p < 0.001; compared with h-eIF4A3-GFP, #p < 0.05, ##p < 0.01, ###p < 0.001. n = 6. **I.** Vulnerable index of plaque were quantified in four groups. Data are presented as mean ± SD. One-way ANOVA, compared with GFP-N.C, *p < 0.05; compared with h-eIF4A3-GFP, #p < 0.05. n = 6. **J.** Vascular reactivity of mice carotid artery rings to acetylcholine (Ach) on phenylephrine (NE) -induced contraction. Data are presented as mean ± SD. Two-way ANOVA, compared with GFP-N.C, *p < 0.05, **p < 0.01, ***p < 0.001; compared with h-eIF4A3-GFP, ###p < 0.001. n = 6.

ELISA results showed that IL-1β and MCP-1 were significantly downregulated in c0030042 compared with the N.C group (Figure 6A-C). Plasma cholesterol, triglyceride, and glucose levels were not significantly different in variable mice groups (Figure S5A-E). In the aorta, c0030042 group showed decreased lipids area, while the h-eIF4A3 group showed increased lipids area compared with the N.C group (Figure 6D-E). We Oil Red O stained cross-section from the thoracic aortas to abdominal aortas, results are shown in Figure S5F-H. Lipids accumulation of h-eIF4A3-GFP group were much higher that of GFP-N.C group; hsa_circ_0030042 decreased lipids deposition. In the aortic root, the relative content of lipids was higher in h-eIF4A3 but lower in c0030042 than in the N.C groups (Figure 6F-G). Relative contents of ICAM/VCAM were higher in h-eIF4A3-GFP but lower in c0030042 than in the N.C groups (Figure 6F, 6H). Conversely, the relative content of collagen was higher in c0030042 but lower in the h-eIF4A3-GFP groups (Figure 6F-G). The content of VSMCs and macrophages was similar in N.C, c0030042, h-eIF4A3-GFP, and c0030042+h-eIF4A3 groups (Figure 6F-G). Vulnerability index, to reflect the instability of AS plaque, was calculated using the following formula 13: (macrophage staining% + lipid staining%) / (VSMC staining% + collagen staining%) in Figure 6I. Compared with the GFP-N.C group, the h-eIF4A3-GFP group showed higher vulnerability index while hsa_circ_0030042 overexpression lowered the vulnerability index. Moreover, the atherogenic effect of h-eIF4A3 on ICAM, VCAM, lipid, and collagen was alleviated by c0030042 (Figure 6F-H). To determine whether endothelial cell-dependent vascular function is affected by hsa_circ_0030042, we measured endothelium-dependent vasodilatation function in the carotid arteries. Ach-induced relaxations were significantly reduced in h-eIF4A3-GFP group, and hsa_circ_0030042 ameliorated the impaired vasorelaxation (Figure 6J). Accordingly, plaque stability was higher in c0030042 but lower in the h-eIF4A3 groups. These results suggest that eIF4A3 augments plaque instability and hsa_circ_0030042 attenuate these effects.

## Discussion

Autophagy poses significant health threats in certain circumstances 24. Abnormal autophagy is implicated in various disorders including cancer, neurodegenerative, and cardiovascular diseases 25, 26. Abnormal autophagy-related beclin1-induced endothelial cell dysfunction and death can promote blood coagulation, enhance platelet adhesion, and promote thrombosis after plaque rupture 5, 27. In addition, abnormal autophagy induces VSMC death, resulting in the secretion of matrix metalloproteinases and degradation of extracellular matrix, which is not conducive to the maintenance of plaque stability 28. FOXO1 modulates autophagic processes and the subsequent cell death 29. Conversely, FOXO1 ablation of ECs in Ldlr^-/-^ mice significantly reduced the expression of cell adhesion molecules (ICAM and VCAM) and reduced the lesion area 6. Further investigations are warranted to identify the role of autophagy in atherosclerosis.

Zhou has reported that the downregulation of circRNA.2837 alleviated sciatic nerve injury by inducing autophagy 30. Meanwhile, circHECTD1 functions as an miR-142 sponge to inhibit TIPARP expression with subsequent inhibition of astrocyte activation via autophagy 31. A few circRNAs are also reportedly involved in atherosclerosis. circANRIL modulates ribosomal RNA maturation and induces apoptosis in atherosclerosis 32. Furthermore, autophagy-related circRNAs reportedly regulate cardiovascular diseases. circRNA ACR attenuated myocardial ischemia by suppressing autophagy via modulation of the Pink1/ FAM65B pathway 14. However, little is known on the role of circRNAs in the stability of atherosclerotic plaques via autophagy. Our data show that, as a highly conserved circRNA, hsa_circ_0030042 is cytoplasmically enriched in endothelial cells and is significantly downregulated in both CHD PBMCs and human atherosclerotic plaques. We illustrated that hsa_circ_0030042 could suppress the overaccumulation of autophagosomes and decrease PS exposure induced by ox-LDL in HUVECs.

eIF4A3 with Y14 and MAGO constitutes the EJC, which accompanies mRNAs during their travel in cells. EJC binds to mRNA via eIF4A3 DEAD-box domain 33, 34. Although EJC is involved in multiple post-transcriptional events, including mRNA transport, translation, and surveillance, EJC deposition is a regulated process. EJC association with particular spliced junctions depends on RNA cis-acting sequences 19. The selectivity of eIF4A3 hints to its complex function. In selenium deficiency, eIF4A3 binding to the selenoprotein mRNA prevents the binding of SECIS binding Protein 2 (SBP2), thereby inhibiting selenoprotein 35. Oncomine demonstrated eIF4A3 overexpression in common malignancies at the transcription level 36. However, no studies on eIF4A3 functions in cardiovascular disease have been reported thus far. In our study, eIF4A3-siRNA decreased mRNA stability and protein expression of beclin1 and FOXO1 in HUVECs. Similarly, hsa_circ_0030042 also decreased beclin1 and FOXO1 mRNA stability and protein expression, while the rebound occurred with upregulation of eIF4A3. However, the effect of hsa_circ_0030042 on beclin1 and FOXO1 was mitigated when its binding site with eIF4A3 was deleted. This indicates that hsa_circ_0030042 regulates autophagy primarily through binding with eIF4A3.

Our results show a novel circRNA that participates in regulating autophagy and plaque stability. hsa_circ_0030042 is significantly downregulated in both CHD PBMCs and human atherosclerotic plaques. PBMCs are more convenient since they are noninvasively acquired, compared to arterial tissues, for clinical tests; hence, our data were sequenced in PBMCs and validated in plaque tissues to facilitate future use of hsa_circ_0030042 in predicting CHD. hsa_circ_0030042 is a novel circRNA which, to the best of our knowledge, has not yet been reported. Its parental gene FOXO1 influences autophagy, apoptosis, oxidative stress, and DNA damage repair; hence, when investigating circRNA function, we initially hypothesised that hsa_circ_0030042 function in atherosclerosis is through its parental gene FOXO1. The subsequent investigation proved this hypothesis. Although the present study shows that hsa_circ_0030042 regulates ox-LDL-induced autophagy by obstructing recruitment of eIF4A3 to beclin1 and FOXO1 mRNA in HUVECs, we should not exclude the possibility that this circRNA may also, directly or indirectly, regulate other vascular cells. With hsa_circ_0030042 overexpression in total aortic plaque tissue, we observed downregulation of autophagy-related proteins at the plaque level and confirmed that hsa_circ_0030042 protects advanced plaque stability by reducing inflammation, decreasing ICAM/VCAM, reducing lipids area, increasing collagen, and improving endothelium-dependent vasodilatation function, *in vivo*. Other hsa_circ_0030042 dependent pathways in the atherogenic model remain to be identified.

## Supplementary Material

Supplementary figures and tables.

## Abbreviations

CHD: coronary heart disease
HUVEC: human umbilical vein endothelial cells
circRNA: circular RNA
ox-LDL: oxidised-low density lipoprotein
eIF4A3: eukaryotic initiation factor 4A-III
EJC: exon junction complex
RBP: RNA-binding protein
FOXO1: forkhead box O1
PS: phosphatidylserine
FISH: fluorescence *in situ* hybridization
RIP: RNA Immunoprecipitation
TG: triglyceride
TC: total cholesterol
ICAM: intercellular cell adhesion molecule I
VCAM: vascular cell adhesion molecule I
