# Supplementary material for: Hsa_circ_0030042 regulates abnormal autophagy and protects atherosclerotic plaque stability by targeting eIF4A3
Source: Theranostics. 2021 Mar 12;11(11):5404–17. doi: 10.7150/thno.48389 (PMC8039966; doi:10.7150/thno.48389)
Supplement: Supplementary file 1 — Supplementary figures and tables. [file thnov11p5404s1.pdf]

Supplementary Materials

Title page: page 1

Materials and Methods: page 2-8

Tables: page 9-13

Figures: page 14-19

Materials and Methods

Collection and treatment of CHD blood and coronary artery samples

70 CHD patients and 30 controls were all selected based on the results of diagnostic coronary angiography. Patients with lung, liver, kidney, immune disease, other heart diseases and other underlying diseases were excluded. Blood samples were collected from patients with CHD who showed no less than 50% coronary stenosis before PCI treatment. The control samples were collected from patients with less than 50% coronary stenosis, based on coronary angiography. All participants were pretreated with conventional-dose Asprin and Clopidogrel before PCI. Age, gender, diabetes history, smoking history, alcohol intake, blood pressure, TG, cholesterol, LDL-C, and HDL-C data for the test subjects and controls were obtained from an electronic medical record system and analysed by *t*-test and Chi-square test. The detailed information were described in Supplementary material Table S3. We separated PBMCs using a PBMCs separation medium (Solarbio, China) and centrifuged twice for total 40 min according to the protocol. Then with the immediate addition of Trizol LS to the PBMCs, all Trizol LS-treated samples were stored at −80°C before sequencing.

Human coronary arteries were collected at the end of the transplant procedure, within 1–2 h of cessation of circulation, constantly under cold ischemic conditions. The coronary arteries with atherosclerotic plaques were matched with normal non-plaque arteries based on histopathological examination in the same individual. were collected at the end of the transplant procedure, within 1–2 h of cessation of circulation, constantly under cold ischemic conditions.

RNA isolation and circRNA sequencing

RNA was isolated according to standardized protocols, and total RNA concentration and quality

were assessed using a NanoDrop ND1000 spectrophotometer (NanoDrop, Wilmington, DE, USA). OD260/OD280 ratios between 1.8 and 2.1 were deemed acceptable. Five micrograms of RNA per sample was used as input material for RNA sample preparation. First, we used an Epicentre Ribo-zero™ rRNA Removal Kit (Epicentre, USA) to obtain rRNA-depleted ribosomal RNAs. rRNA-depleted RNAs were further treated with RNase R (Epicentre, USA). Subsequently, sequencing libraries were generated from the rRNA-depleted and RNase R digested RNAs using an NEBNext® Ultra™ Directional RNA Library Prep Kit for Illumina® (NEB, USA) following the manufacturer's recommendations. Briefly, the RNA was fragmented using divalent cations at an elevated temperature in NEBNext First Strand Synthesis Reaction Buffer. First-strand cDNA was synthesized using random hexamer primers and M-MuLV Reverse Transcriptase (RNaseH−). Second-strand cDNA synthesis was then completed using DNA Polymerase I and RNase H. In the reaction buffer, dNTPs with dTTP were replaced by dUTP. Remaining overhangs were converted into blunt ends via exonuclease/polymerase activity. After adenylation of the 3' ends of DNA fragments, NEBNext Adaptors with hairpin loop structures were ligated to prepare for hybridization. To select cDNA fragments 150-200 bp in length, the library fragments were purified using an AMPure XP system (Beckman Coulter, Beverly, USA). Then 3 μl of USER Enzyme (NEB, USA) was allowed to react with size-selected, adaptor-ligated cDNA at 37°C for 15 min, followed by 5 min at 95°C before PCR. PCR was performed with Phusion High-Fidelity DNA polymerase, Universal PCR primers, and Index (X) Primers. Finally, the library was purified (AMPure XP system) and then qualified using an Agilent Bioanalyzer 2100 system. Clustering of the index-coded samples was performed on a cBot Cluster Generation System with a HiSeq PE Cluster Kit v4 cBot (Illumina) according to the manufacturer's instructions. After cluster generation, the library

preparations were sequenced on an Illumina HiSeq 2500 platform, and 125-bp paired-end reads were generated. We defined a circRNA expressed or not by junction reads with software CIRI2 (version 2.0.5), CIRI AS (version 1.2) and find_circ (version 1.2). We defined the statistical criteria with software DESeq2 (version1.10.1) for significant differently expressed circRNA as having a p-value < 0.05 and a fold change ≥ 2.0 or ≤ 0.5

HUVECs culture and treatment

HUVECs were acquired from ScienCell Research Laboratories (Carlsbad, CA, USA) and cultured in endothelial cell medium (ECM) (ScienCell, Carlsbad, CA) with 5% fetal bovine serum (FBS), 1% endothelial cell growth supplement and 1% penicillin/streptomycin at 37 °C in 5% CO2. Passage 4-8 were used for experiment. 100ug/ml human ox-LDL (Yiyuan Biotechnologies, Guangzhou, China) was added into cell culture according to experimental requirements.

Transfection of stubRFP-sensGFP-LC3 adenovirus

To observe the autophagy of HUVECs, stubRFP-sensGFP-LC3 adenovirus (Genechem, Shanghai, China) were transfected into cells according to the manufacturer's instructions. Following transfection for 72 h, HUVECs were stimulated with 100ng/ml ox-LDL for 0 h, 3 h and 24 h. After fixed with 4% formaldehyde, the autophagosomes were photographed using confocal laser scanning microscopy.

Northern blot

Northern blot was performed as others described. In brief, the samples were run on a 1% formaldehyde-polyacrylamide-urea gel, transferred to positively charged Hybond N+ membranes (Amersham) followed by cross-linking through UV irradiation. The membranes were subjected to hybridization with 100 pmol 3'-digoxigenin (DIG)-labeled probe for hsa_circ_0030042 overnight

at 50°C. hsa_circ_0030042 probe was synthesized with PCR DIG Probe Synthesis Kit (Roche). The detection was performed using a DIG High Prime DNA Labeling and Detection Starter Kit II (Roche) according to the protocol. The forward primer for hsa_circ_0030042 probe sequence was 5'-agtgacttggatggcatgtt-3'; the reverse primer was 5'-tctggattgagcatccaccaaga-3'. DIG-labeled GAPDH probe was used as control, and its forward primer was 5′-aatcccatcaccatcttcc-3'; the reverse primer was 5'-catcacgccacagtttcc-3'.

Western blot

HUVECs and aortic tissue samples were lysed using RIPA buffer (Solarbio, Beijing, China) for 20 min and collected by centrifugation at 12000 rpm for 10 min at 4°C. Nuclear and cytoplasmic proteins were extracted (Extraction Reagents, Thermo, USA) as needed according to the attached protocol. Equal amounts of proteins and pre-stained protein ladder (Thermo Fisher Scientific) were separated through 12% SDS-PAGE gels (TGX FastCast Acrylamide Kit, Bio-Rad, USA) Then, proteins were transferred to methanol-activated polyvinylidene fluoride membranes with a 0.2 μm pore size (Millipore, Billerica, MA, USA), and incubated with primary antibodies overnight at 4 °C. The membranes were incubated with secondary antibodies (ProteinTech, Rosemont, Penn., USA) the next day for 1 h at room temperature. Bands with antigen-antibody complexes were visualized using Immobilon ECL substrate (Millipore, Billerica, MA, USA), and blots were imaged with a LAS-4000 luminescent image analyser (Fujifilm USA, Valhalla, NY, USA). Protein expression was quantified using Adobe Photoshop CS6 (Adobe Systems, San Jose, CA, USA), normalized to GAPDH expression in each sample, and expressed as a percentage of the control. The anti-FOXO1 antibody (1:1000, 2880, CST), anti-beclin1 antibody (1:1000, ab207612), anti-LC3B antibody (1:2000, ab192890), anti-eIF4A3 antibody (1:1000, ab180573) and anti-GAPDH antibody (1:1000,

5174, CST) were used in this study.

RNA and gDNA extraction and quantitative real-time PCR

Total RNA was extracted from HUVECs or tissues using TRIzol reagent (Ambion, Life Technologies, Waltham, MA, USA). The RNA was treated with rRNA depletion (GeneRead, QIAGEN, Germany) or linear RNA depletion (Epicentre, Illumina, Germany) as required. The product was reverse-transcribed into cDNA using a PrimeScript RT Reagent Kit (TakaRa Biotechnology, Dalian, China). The cDNA (1 ng) was subjected to qPCR using SYBR Green (TakaRa) for the relative quantification of mRNA expression. Quantification was accomplished using the 2-ΔΔCt method. GAPDH was used to normalize mRNA levels. The hsa_circ_0030042 divergent primer 1: 5'- ctttgacaatgtgttgccca-3' (forward); 5'-aggagatttcccgctcttg-3' (reverse). The hsa_circ_0030042 divergent primer 2: 5'-tggatggagatacattggatt-3' (forward); 5'-attgagcatccaccaagaac-3' (reverse). The beclin1 forward primer: 5'-aatggtggctttcctggact-3'; the reverse primer: 5'-catccatcctgtagggaagacaa-3'. The FOXO1 forward primer: 5'-cgcttggactgtgacatgga-3'; the reverse primer: 5'-aatgtagcctgctcactaactc-3'.

Total gDNA was extracted from HUVECs using Genomic DNA Extraction Kit (TAKARA) according to the procedure. The hsa_circ_0030042 convergent forward primer: 5'-ggcagccaggcatctcataa-3', the convergent reverse primer: 5'-ttgggtcaggcggttcatac-3'. The homo-GAPDH convergent forward primer: 5'-aagaaggtggtgaagcaggc-3'; the convergent reverse primer: 5'-gtcaaaggtggaggagtggg-3'. The homo-GAPDH divergent forward primer: 5'-agaaggctggggctcatttg-3'; the divergent reverse primer: 5'-tcgccccacttgattttgga-3'.

Electron Microscopy

Stable hsa_circ_0030042 overexpression HUVECs (c0030042) and empty vector transfected

HUVECs (circ-N.C) were transiently transfected with eIF4A3 siRNA for 48 h and 100ug/ml ox-LDL treated 24h. Then the cells were collected, 1,000 rpm centrifuge 5 min, the supernatant was discarded, 1 mL PBS resuspended, centrifuged 10 min, the supernatant was discarded, fixed with 2.5% glutaraldehyde, and then the the cells were embedded in spur resin after dehydration. Thin sections were cut on a Reichert Ultracut E microtome. Sectioned grids were stained with saturated solution of uranyl acetate and lead citrate. Sections were examined at 80 kV with a Hitachi transmission electron microscope.

Indirect Immunofluorescence Assay and Confocal Microscopy

circ-N.C and c0030042 group cells were grown on cover slips in 24-well plates. Upon reaching 70–80% confluence, cells were fixed in 4% paraformaldehyde for 30 min at room temperature and washed with PBS. Subsequently, permeabilized with PBS containing 0.5% Triton-X-100 for 15 min and blocked with PBS containing 5% bovine serum albumin for 1 h at room temperature. Then, samples were incubated with anti-eIF4A3 antibody (1:500, ab180573) for 4°C overnight, followed by incubation with anti-rabbit IgG Alexa Fluor 594-conjugated antibody for 1 h, and cells nuclei were visualized with 4′,6-diamidino-2-phenylindole (DAPI, Invitrogen). All fluorescence images were acquired on an Olympus confocal microscope.

Flow Cytometry

Phosphotidylserine (PS) exposure of ox-LDL treated cells were analyzed using an Annexin V PE/7-Amino-Actinomycin (7-AAD) Apoptosis Detection Kit (BD Pharmingen, CA, USA) according to the protocol. Unstained control cells, cells stained with PE Annexin V only, and cells stained with 7-AAD only were used to set up compensation and to define quadrants. Apoptotic cells were examined within 1 h, and the percentage of PS positive cells (right quadrant) was measured using

FlowJo software (Tree Star, Ashland, OR, USA).

Actinomycin D treatment

To block transcription, cell culture medium was added with 10 ug/ml Actinomycin D (Sigma-Aldrich, St. Louis, MO, USA) in 0h, 3h, 6h, 9h and 12h. After treatment with Actinomycin D for different time points, the remaining of mRNA was assessed using qRT-PCR.

Enzyme-Linked Immunosorbent Assay (ELISA)

The level of IL-1β in mice plasma was determined using a mouse IL-1β ELISA Kit (ab197742, abcam, UK). The level of IL-6 in mice plasma was assessed using a mouse IL-6 ELISA Kit (ab222503). The level of MCP-1 in mice plasma was tested using a mouse MCP-1 ELISA Kit (R&D system, Minneapolis, MN, USA). All kits were used according to the manufacturer's instructions. The OD value was recorded at 450 nm (with reference of 570 nm) in an ELISA plate reader.

Table S1: Significant altered circRNA profile in CHD PBMCs

Sequencing ID	circBase ID	log2Fold Change	pval	padj	significant	trend	chrom	txStart	txEnd	length	GeneName	Catalog
hg38_circ_0052138		3.1318	3.06E-12	1.43E-09	TRUE	up	chr19	39883304	39896143	1000	FCGBP	exonic
hg38_circ_0081334	hsa_circ_0119359	3.0972	9.32E-10	1.51E-07	TRUE	up	chr2	232851276	232851449	173	GIGYF2	antisense
hg38_circ_0114298		2.9897	4.01E-07	2.08E-05	TRUE	up	chr6	32661966	32757883	100	HLA-DQB1	sense overlapping
hg38_circ_0050204		2.919	1.03E-09	1.62E-07	TRUE	up	chr19	12792306	12792484	500	HOOK2	intronic
hg38_circ_0052139		2.8563	9.14E-06	0.000257	TRUE	up	chr19	39885672	39899697	1000	FCGBP	exonic
hg38_circ_0085746	hsa_circ_0006251	2.719	1.40E-13	1.15E-10	TRUE	up	chr2	74046277	74048411	2134	TET3	exonic
hg38_circ_0057649	hsa_circ_0110941	2.6958	4.57E-06	0.000147	TRUE	up	chr1	1637761	1704655	66894	CDK11B	sense overlapping
hg38_circ_0057643		2.6933	4.73E-10	8.77E-08	TRUE	up	chr1	1636332	1703269	100	CDK11B	sense overlapping
hg38_circ_0074503	hsa_circ_0001232	2.5501	6.67E-09	7.67E-07	TRUE	up	chr22	39015594	39021536	5942	AL022318.4	intronic
hg38_circ_0052140		2.4768	4.28E-05	0.000864	TRUE	up	chr19	39885990	39902124	1000	FCGBP	exonic
hg38_circ_0006441		2.4594	4.30E-06	0.00014	TRUE	up	chr11	105030329	105066245	100	CASP1	sense overlapping
hg38_circ_0036776		2.3949	5.30E-23	1.09E-18	TRUE	up	chr16	31362610	31410524	100	ITGAX	sense overlapping
hg38_circ_0113938		2.3868	5.18E-15	8.89E-12	TRUE	up	chr6	29945450	30009367	100	HCG9	sense overlapping
hg38_circ_0073043	hsa_circ_0062287	2.3855	0.00023315	0.003293	TRUE	up	chr22	20052464	20056013	3549	TANGO2	exonic
hg38_circ_0057646	hsa_circ_0110940	2.363	1.36E-05	0.000349	TRUE	up	chr1	1637407	1704344	66937	CDK11B	sense overlapping
hg38_circ_0057551		2.3542	6.04E-07	2.94E-05	TRUE	up	chr1	161548420	161626402	100	FCGR3B	sense overlapping
hg38_circ_0009842		2.3409	0.0002752	0.003761	TRUE	up	chr11	48027270	48027508	500	PTPRJ	intronic
hg38_circ_0013083		2.2578	0.00019466	0.002845	TRUE	up	chr11	9248409	9248643	100	DENND5A	antisense
hg38_circ_0056414	hsa_circ_0004482	2.0927	0.0013975	0.012973	TRUE	up	chr1	151262160	151264028	1868	PSMD4	exonic
hg38_circ_0035431		2.0904	0.0014127	0.013061	TRUE	up	chr16	1744583	1744762	100	MAPK8IP3	antisense
hg38_circ_0030223	hsa_circ_0103395	2.0502	0.00014066	0.002234	TRUE	up	chr15	39582470	39582680	210	THBS1	sense overlapping
hg38_circ_0056093	hsa_circ_0000004	2.0306	1.31E-07	8.32E-06	TRUE	up	chr1	1495484	1529331	33847	ATAD3B	sense overlapping
hg38_circ_0114300		2.0061	0.0014718	0.013445	TRUE	up	chr6	32664797	32759131	100	HLA-DQB1	sense overlapping

hg38_circ_0056967	hsa_circ_0014624	1.9942	0.0023522	0.019066	TRUE	up	chr1	155764999	155766709	1710	GON4L	exonic
hg38_circ_0060201		1.9798	0.0012041	0.01164	TRUE	up	chr1	207563863	207578203	1000	CR1	exonic
hg38_circ_0113778		1.9489	6.85E-06	0.000203	TRUE	up	chr6	26407670	26444304	100	BTN3A1	sense overlapping
hg38_circ_0010305	hsa_circ_0022392	1.9392	0.0014126	0.013061	TRUE	up	chr11	61862971	61863786	815	FADS2	exonic
hg38_circ_0124959	hsa_circ_0083377	1.8628	6.89E-06	0.000204	TRUE	up	chr8	13088486	13088704	218	DLC1	exonic
hg38_circ_0119325		1.8091	0.0053904	0.034593	TRUE	up	chr7	142791693	142796610	100	TRBJ2-1	sense overlapping
hg38_circ_0010405		1.8003	0.00087022	0.009096	TRUE	up	chr11	62539659	62539872	500	AHNAK	intronic
hg38_circ_0119326		1.7969	3.46E-11	1.11E-08	TRUE	up	chr7	142791693	142796895	100	TRBJ2-1	sense overlapping
hg38_circ_0052137	hsa_circ_0109623	1.7821	0.00019458	0.002845	TRUE	up	chr19	39877662	39893527	15865	FCGBP	exonic
hg38_circ_0051386		1.7679	0.0050955	0.033166	TRUE	up	chr19	3197909	3198222	100	NCLN	antisense
hg38_circ_0052591		1.7416	2.97E-06	0.000106	TRUE	up	chr19	45227661	45228266	1000	EXOC3L2	exonic
hg38_circ_0004576		1.6917	0.0097274	0.053075	FALSE	up	chr10	70123404	70124097	1000	AIFM2	exonic
hg38_circ_0042152		1.6878	5.13E-06	0.000161	TRUE	up	chr17	3935191	3936469	1000	ATP2A3	exonic
hg38_circ_0054091		1.6846	0.0052026	0.033692	TRUE	up	chr19	7643749	7643988	500	STXBP2	intronic
hg38_circ_0054092		1.6846	0.0052026	0.033692	TRUE	up	chr19	7643756	7643995	100	STXBP2	antisense
hg38_circ_0054093		1.6846	0.0052026	0.033692	TRUE	up	chr19	7643774	7644013	100	STXBP2	antisense
hg38_circ_0105998		1.6818	6.01E-05	0.001128	TRUE	up	chr5	176891036	176893088	100	HK3	sense overlapping
hg38_circ_0057873		1.6691	0.005902	0.037215	TRUE	up	chr1	1676063	1738448	100	SLC35E2	sense overlapping
hg38_circ_0000077		1.6684	0.0033533	0.02474	TRUE	up	chr10	100529532	100529768	500	HIF1AN	intronic
hg38_circ_0018333	hsa_circ_0000400	1.6669	1.17E-10	2.83E-08	TRUE	up	chr12	49131297	49186833	55536	TUBA1A	sense overlapping
hg38_circ_0099741		1.6607	0.00035954	0.004608	TRUE	up	chr4	3518033	3525051	1000	LRPAP1	exonic
hg38_circ_0004146		1.6563	0.0060082	0.037665	TRUE	up	chr10	67894973	67895137	500	SIRT1	intronic
hg38_circ_0050770	hsa_circ_0050001	1.6466	0.0013571	0.012649	TRUE	up	chr19	17530754	17532388	1634	FAM129C	exonic
hg38_circ_0053488		1.6266	0.0081784	0.047082	TRUE	up	chr19	54221827	54241127	100	LILRB3	sense overlapping
hg38_circ_0056768	hsa_circ_0000135	1.6158	0.013197	0.066006	FALSE	up	chr1	155212384	155232845	20461	MTX1	sense overlapping
hg38_circ_0031586	hsa_circ_0035271	1.6113	0.013751	0.067835	FALSE	up	chr15	50958491	50958794	303	AP4E1	exonic
hg38_circ_0057816	hsa_circ_0000006	1.6017	4.62E-09	5.57E-07	TRUE	up	chr1	1669663	1734835	65172	SLC35E2	sense overlapping

hg38_circ_0135799	hsa_circ_0001947	-3.8854	1.21E-15	2.86E-12	TRUE	down	chrX	148661907	148662768	861	AFF2	exonic
hg38_circ_0121582	hsa_circ_0001707	-3.8139	9.17E-13	4.96E-10	TRUE	down	chr7	48502125	48502552	427	ABCA13	intronic
hg38_circ_0135798	hsa_circ_0091669	-3.5986	1.56E-11	5.62E-09	TRUE	down	chrX	148651998	148662768	10770	AFF2	exonic
hg38_circ_0079406	hsa_circ_0003915	-3.4907	1.06E-10	2.69E-08	TRUE	down	chr2	199368604	199433514	64910	SATB2	exonic
hg38_circ_0098952	hsa_circ_0001460	-3.3323	2.45E-13	1.72E-10	TRUE	down	chr4	177353307	177360677	7370	NEIL3	exonic
hg38_circ_0099921	hsa_circ_0126249	-3.3155	9.43E-09	1.02E-06	TRUE	down	chr4	38790069	38791231	1162	TLR1	sense overlapping
hg38_circ_0022581	hsa_circ_0030042	-3.1674	2.02E-08	1.84E-06	TRUE	down	chr13	40559508	40560860	1352	FOXO1	exonic
hg38_circ_0115660	hsa_circ_0005893	-3.1646	1.45E-07	9.04E-06	TRUE	down	chr6	5396632	5431172	34540	FARS2	sense overlapping
hg38_circ_0004176	hsa_circ_0093906	-3.1486	2.61E-08	2.19E-06	TRUE	down	chr10	67966682	68014186	47504	HERC4	exonic
hg38_circ_0123175	hsa_circ_0135105	-3.1455	1.15E-07	7.53E-06	TRUE	down	chr7	92517275	92518255	980	PEX1	exonic
hg38_circ_0120968	hsa_circ_0079813	-3.0885	1.09E-07	7.21E-06	TRUE	down	chr7	33351218	33388144	36926	BBS9	exonic
hg38_circ_0095191	hsa_circ_0066187	-3.0799	1.02E-08	1.08E-06	TRUE	down	chr3	53497151	53501720	4569	CACNA1D	exonic
hg38_circ_0098239	hsa_circ_0125534	-3.0591	1.58E-06	6.38E-05	TRUE	down	chr4	152411302	152469988	58686	FBXW7	sense overlapping
hg38_circ_0062467	hsa_circ_0112791	-3.0025	3.92E-06	0.00013	TRUE	down	chr1	243637610	243695716	58106	AKT3	exonic
hg38_circ_0137512	hsa_circ_0001924	-2.9883	1.42E-09	2.09E-07	TRUE	down	chrX	65051461	65075912	24451		intergenic
hg38_circ_0110949		-2.9672	4.98E-06	0.000157	TRUE	down	chr6	127829475	127901081	1000	THEMIS	exonic
hg38_circ_0064279	hsa_circ_0113258	-2.9525	5.97E-06	0.000183	TRUE	down	chr1	38441202	38442939	1737		intergenic
hg38_circ_0120829	hsa_circ_0134091	-2.9501	5.05E-07	2.54E-05	TRUE	down	chr7	30550635	30574881	24246	AC005154.1	sense overlapping
hg38_circ_0130736	hsa_circ_0138147	-2.9451	1.59E-07	9.53E-06	TRUE	down	chr9	129978464	129994958	16494	FNBP1	exonic
hg38_circ_0049887	hsa_circ_0049329	-2.9132	4.43E-07	2.27E-05	TRUE	down	chr19	10772478	10786706	14228	DNM2	exonic
hg38_circ_0127573		-2.8874	1.22E-06	5.19E-05	TRUE	down	chr8	66572481	66602523	1000	MYBL1	exonic
hg38_circ_0022874	hsa_circ_0100578	-2.8693	2.58E-07	1.45E-05	TRUE	down	chr13	45336535	45337388	853	TPT1	sense overlapping
hg38_circ_0031488	hsa_circ_0103771	-2.8595	1.46E-08	1.42E-06	TRUE	down	chr15	50586391	50592626	6235	TRPM7	exonic
hg38_circ_0025991	hsa_circ_0101893	-2.8371	3.43E-06	0.000119	TRUE	down	chr14	45246742	45247377	635	MIS18BP1	exonic
hg38_circ_0095798	hsa_circ_0066529	-2.8294	1.08E-06	4.71E-05	TRUE	down	chr3	71015548	71047095	31547	FOXP1	exonic
hg38_circ_0089028	hsa_circ_0122078	-2.7969	1.47E-05	0.00037	TRUE	down	chr3	136443286	136502779	59493	STAG1	exonic
hg38_circ_0127744	hsa_circ_0001807	-2.7781	2.23E-08	1.97E-06	TRUE	down	chr8	67251298	67259926	8628	ARFGEF1	exonic

hg38_circ_0030754	hsa_circ_0006297	-2.7756	5.35E-06	0.000167	TRUE	down	chr15	42827927	42878684	50757	TTBK2	exonic
hg38_circ_0047155	hsa_circ_0107922	-2.7722	2.38E-07	1.35E-05	TRUE	down	chr18	12999420	13030608	31188	CEP192	exonic
hg38_circ_0003136	hsa_circ_0093547	-2.7691	1.75E-08	1.66E-06	TRUE	down	chr10	32451591	32481782	30191	CCDC7	sense overlapping
hg38_circ_0127168	hsa_circ_0136720	-2.7321	1.38E-05	0.000353	TRUE	down	chr8	51831443	51861246	29803	PCMTD1	exonic
hg38_circ_0090544	hsa_circ_0001356	-2.7257	1.01E-05	0.000277	TRUE	down	chr3	160413472	160414517	1045	SMC4	exonic
hg38_circ_0095699	hsa_circ_0001319	-2.7183	6.98E-07	3.29E-05	TRUE	down	chr3	69027899	69028295	396	TMF1	exonic
hg38_circ_0116787	hsa_circ_0132617	-2.6917	1.41E-07	8.84E-06	TRUE	down	chr6	89083752	89084643	891	PNRC1	sense overlapping
hg38_circ_0106454	hsa_circ_0075370	-2.6851	4.09E-07	2.12E-05	TRUE	down	chr5	180808647	180809076	429	MGAT1	exonic
hg38_circ_0090764	hsa_circ_0001274	-2.679	3.59E-06	0.000122	TRUE	down	chr3	17009673	17014911	5238	PLCL2	exonic
hg38_circ_0022154	hsa_circ_0029853	-2.6589	3.13E-07	1.71E-05	TRUE	down	chr13	28256291	28281379	25088	PAN3	exonic
hg38_circ_0054602		-2.658	1.96E-05	0.000464	TRUE	down	chr1	103565434	103621893	100	AMY2B	sense overlapping
hg38_circ_0098048	hsa_circ_0125480	-2.6563	2.61E-06	9.66E-05	TRUE	down	chr4	148152468	148154901	2433	NR3C2	exonic
hg38_circ_0082078	hsa_circ_0119551	-2.6358	2.14E-05	0.000497	TRUE	down	chr2	24510968	24564430	53462	NCOA1	sense overlapping
hg38_circ_0079201	hsa_circ_0057551	-2.622	4.65E-06	0.000149	TRUE	down	chr2	195680031	195683906	3875	SLC39A10	exonic
hg38_circ_0001528	hsa_circ_0000264	-2.6208	5.08E-07	2.55E-05	TRUE	down	chr10	124038514	124046724	8210	CHST15	exonic
hg38_circ_0106420		-2.6201	1.21E-05	0.000317	TRUE	down	chr5	180529274	180542008	500	CNOT6	intronic
hg38_circ_0033670	hsa_circ_0104611	-2.6188	1.71E-06	6.79E-05	TRUE	down	chr15	77133004	77133750	746	PEAK1	exonic
hg38_circ_0022428	hsa_circ_0100273	-2.6105	5.61E-06	0.000174	TRUE	down	chr13	32535768	32537027	1259	N4BP2L2	exonic
hg38_circ_0065795	hsa_circ_0012721	-2.604	7.54E-08	5.26E-06	TRUE	down	chr1	58527260	58539310	12050	OMA1	exonic
hg38_circ_0031444		-2.5961	2.90E-06	0.000104	TRUE	down	chr15	50458999	50459162	900	USP8	exonic
hg38_circ_0137903	hsa_circ_0140608	-2.5912	3.98E-05	0.000812	TRUE	down	chrX	77633206	77656653	23447	ATRX	exonic
hg38_circ_0070407	hsa_circ_0115396	-2.5771	2.91E-05	NA	NA	down	chr20	49264715	49271750	7035	ZNFX1	exonic
hg38_circ_0127171	hsa_circ_0136721	-2.5768	7.87E-06	0.000226	TRUE	down	chr8	51845660	51861246	15586	PCMTD1	exonic

Table S1 Top 50 significantly upregulated and 50 down regulated in CHD PBMCs were shown in details. The red color represented the up-regulated circRNAs. The green color represented the down-regulated circRNAs in CHD. The blue color showed the selected exonic CHD related circRNA, hsa_circ_0030042. Differential expression analysis between the two groups was performed using DESeq2. The adjusted p-value (padj) is the p-value adjusted for multiple testing using Benjamini-Hochberg to estimate the false discovery rate (FDR). circRNAs with a padj < 0.05 and a fold change ≥2.0 or ≤0.5 were considered differentially expressed.

Table S2: Interaction probabilities of three RBPs to hsa_circ_0030042

RBP	Tags of RBPs matching flanking regions on hsa_circ_0030042	RF classifier	SVM classifier
**AGO2**	8	0.75	0.9
**eIF4A3**	6	0.75	0.83
**HuR**	3	0.8	0.75

Table S2: Detailed information of RPISeq (version 1.0) predicting the probabilities of three RBPs combine with hsa_circ_0030042.

Table S3: Basic information of selected coronary heart disease patients and controls.

Variables<br>Mean ± SD or n (percentage)	ctrl<br>n = 30	CHD<br>n = 70	P
**Stenosis of the left main coronary trunk or maximum stenosis in a major epicardial artery (%)**	16.67±17.29	79.21±17.40	< 0.0001
**TC (mmol/l)**	4.148±0.17	4.040±0.13	ns
**LDL-C (mmol/l)**	2.456±0.12	2.597±0.12	ns
**HDL-C (mmol/l)**	1.303±0.06	1.198±0.03	ns
**Age (years)**	57.57±1.39	60.99±1.18	ns
**Gender (M/FM)**	16(53)/14(47)	49(70)/21(30)	ns
**Diabetes (yes/no)**	4(13)/26(87)	16(23)/54(77)	ns
**Smoking history (yes/no)**	10(33)/20(67)	37(53)/33(47)	ns
**Family history of CHD (yes/no)**	4(13)/26(87)	19(27)/51(73)	ns
**Alcohol intake history (yes/no)**	9(30)/21(70)	26(37)/44(63)	ns
**Other underlying diseases**	no	no	ns
**Pharmacological therapy**	Asprin and Clopidogrel	Asprin and Clopidogrel	ns

Table S3: Detailed information of coronary heart disease patients and controls where PBMCs acquired. TC: total cholesterol, LDL-C: low-density lipoprotein cholesterol, HDL-C: high-density lipoprotein cholesterol

Supplementary Figure 1:

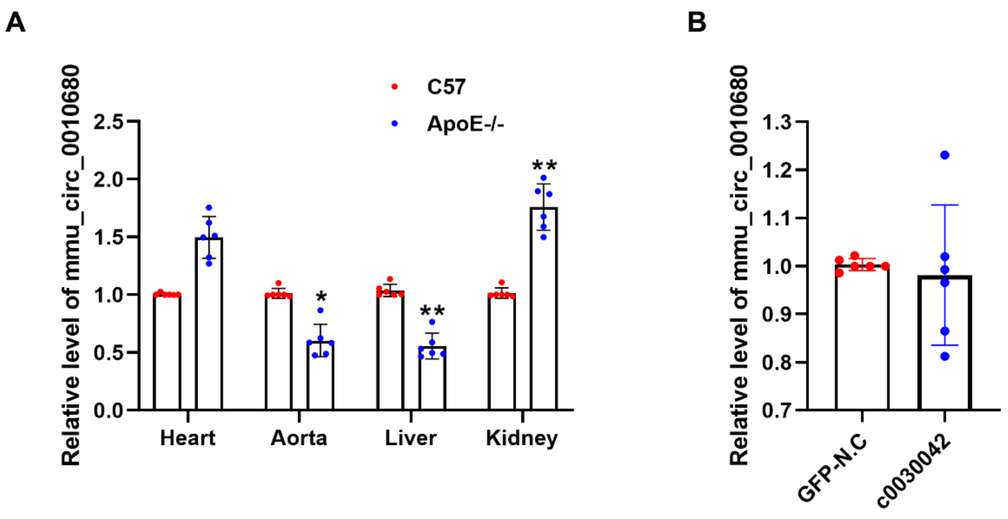

Supplementary Figure 1 qRT-PCR validated mmu_circ_0010680 in 3-month-old C57BL/6 and ApoE-/- mice. Data are presented as mean ± SD. Student's t-test. *p < 0.05, **p < 0.01. n = 6 pairs.

Supplementary Figure 2:

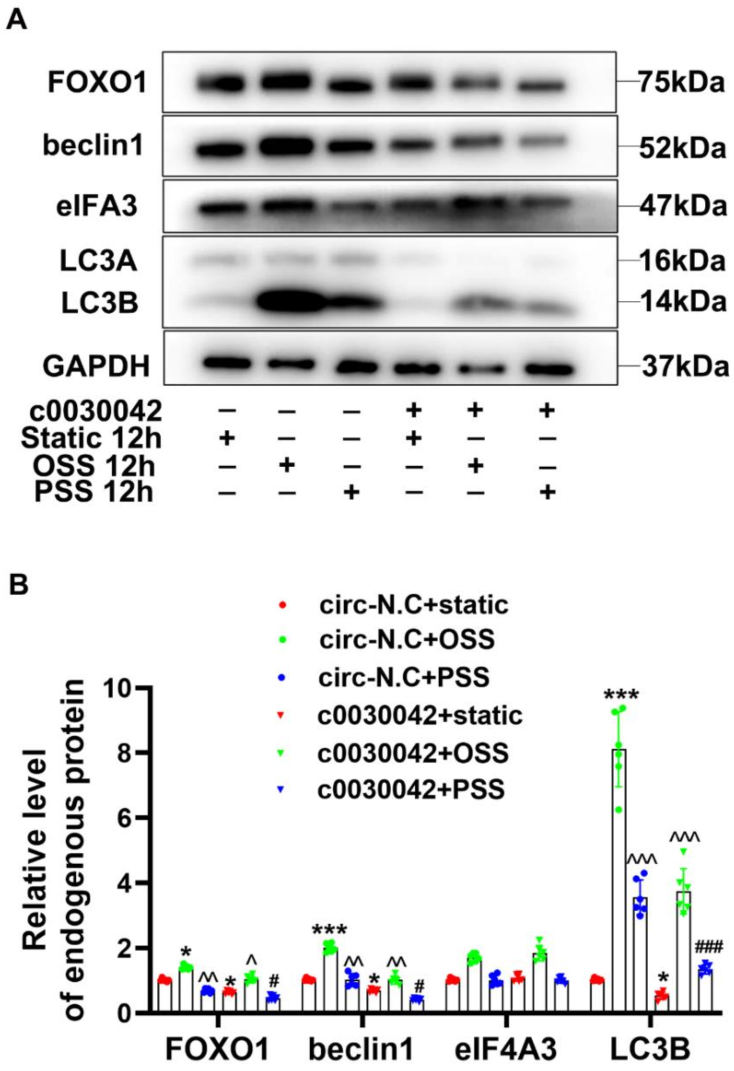

Supplementary Figure 2 Western blot for quantifying protein levels in 12h shear stress-stimulated c0030042 and circ-N.C (HCAECs). Two-way ANOVA. Compared with circ-N.C + static, *p < 0.05, ***p < 0.001; compared with circ-N.C+OSS, ^p < 0.05, ^^p < 0.01, ^^^p < 0.001; compared with circ-N.C+PSS, #p < 0.05, ###p < 0.001. n = 6. PSS, unidirectional pulsatile shear stress, 12 dyne/cm2, OSS, oscillatory shear stress, 0±4 dyne/cm2. Data are presented as mean ± SD.

Supplementary Figure 3:

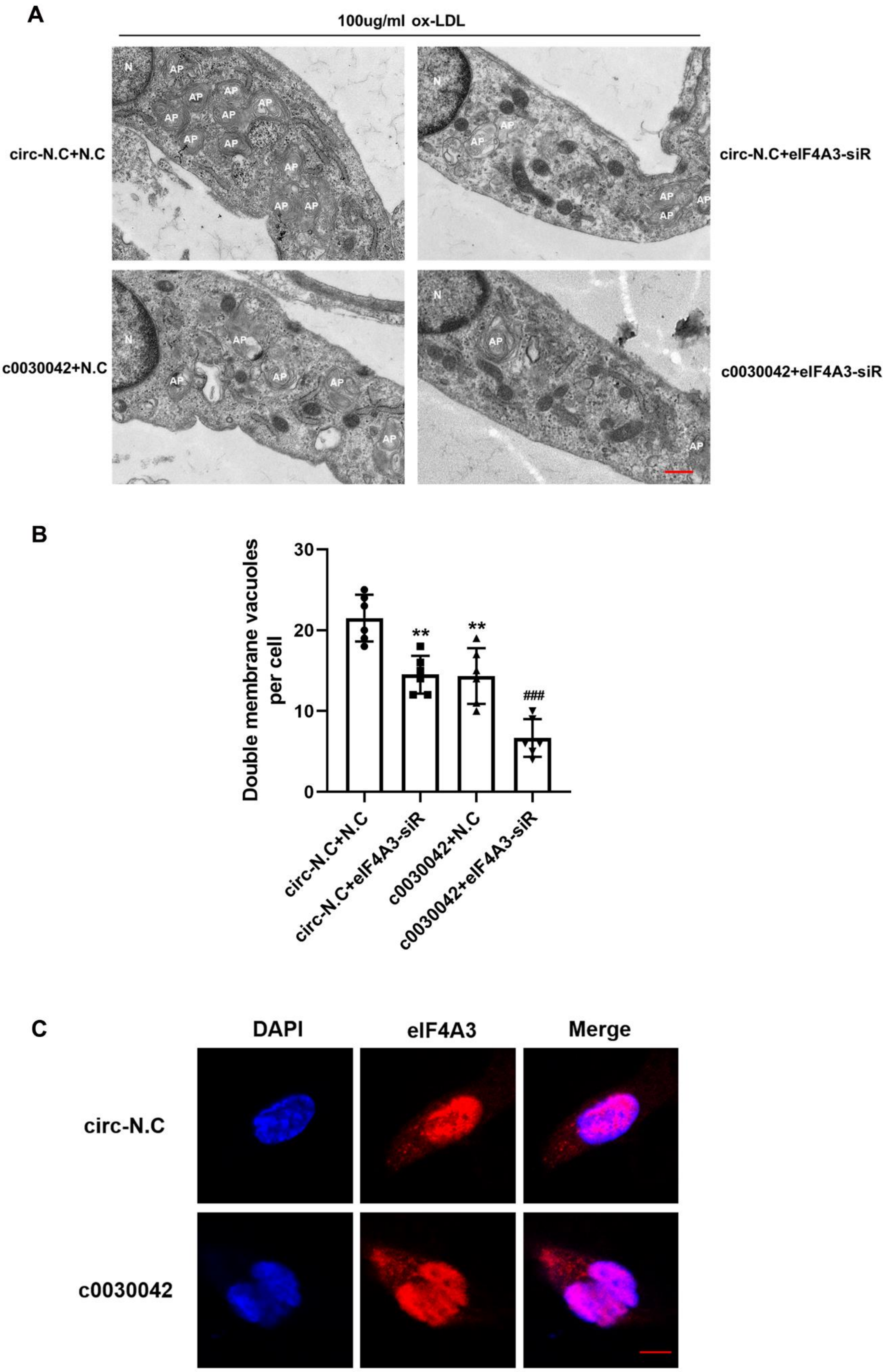

Supplementary Figure 3 eIF4A3 depression could further decrease the autophagic vacuoles in both c0030042 and circ-N.C group. **A.** Autophagy by Electron Microscope (×15000). Stable hsa_circ_0030042 overexpression HUVECs (c0030042) and empty vector transfected HUVECs (circ-N.C) were transiently transfected with eIF4A3 siRNA or N.C for 48 h and 100ug/ml ox-LDL treated 24h. Autophagic vacuoles were indicated. Scale bar, 5μm. **B.** Each group counts six cells and showing the average number of double membrane vacuoles per cell. The data were expressed as mean ± SD. One-way ANOVA. Compared with circ-N.C+N.C, **$p < 0.01$; compared with c0030042+N.C, ###$p < 0.001$). n = 6. **C.** Immunofluorescence confocal results of eIF4A3 localization in circ-N.C and c0030042 group. Scale bar, 10μm.

Supplementary Figure 4:

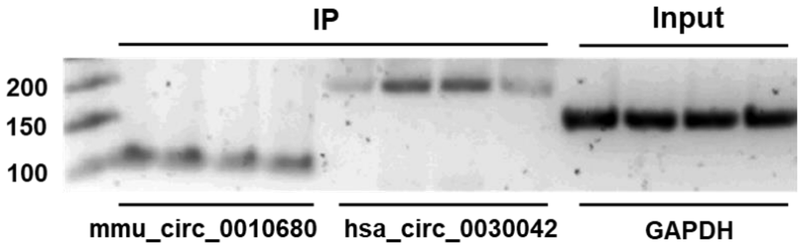

Supplementary Figure 4 Murine eIF4A3 could interact with mmu_circ_0010680 and hsa_circ_0030042. RNA immunoprecipitation of eIF4A3 from mice with 6-weeks c0030042-lentivirus transfection. hsa_circ_0030042, mmu_circ_0010680 and GAPDH qRT-PCR products were determined by agarose gel electrophoresis. n=5 mice of each sample.

Supplementary Figure 5:

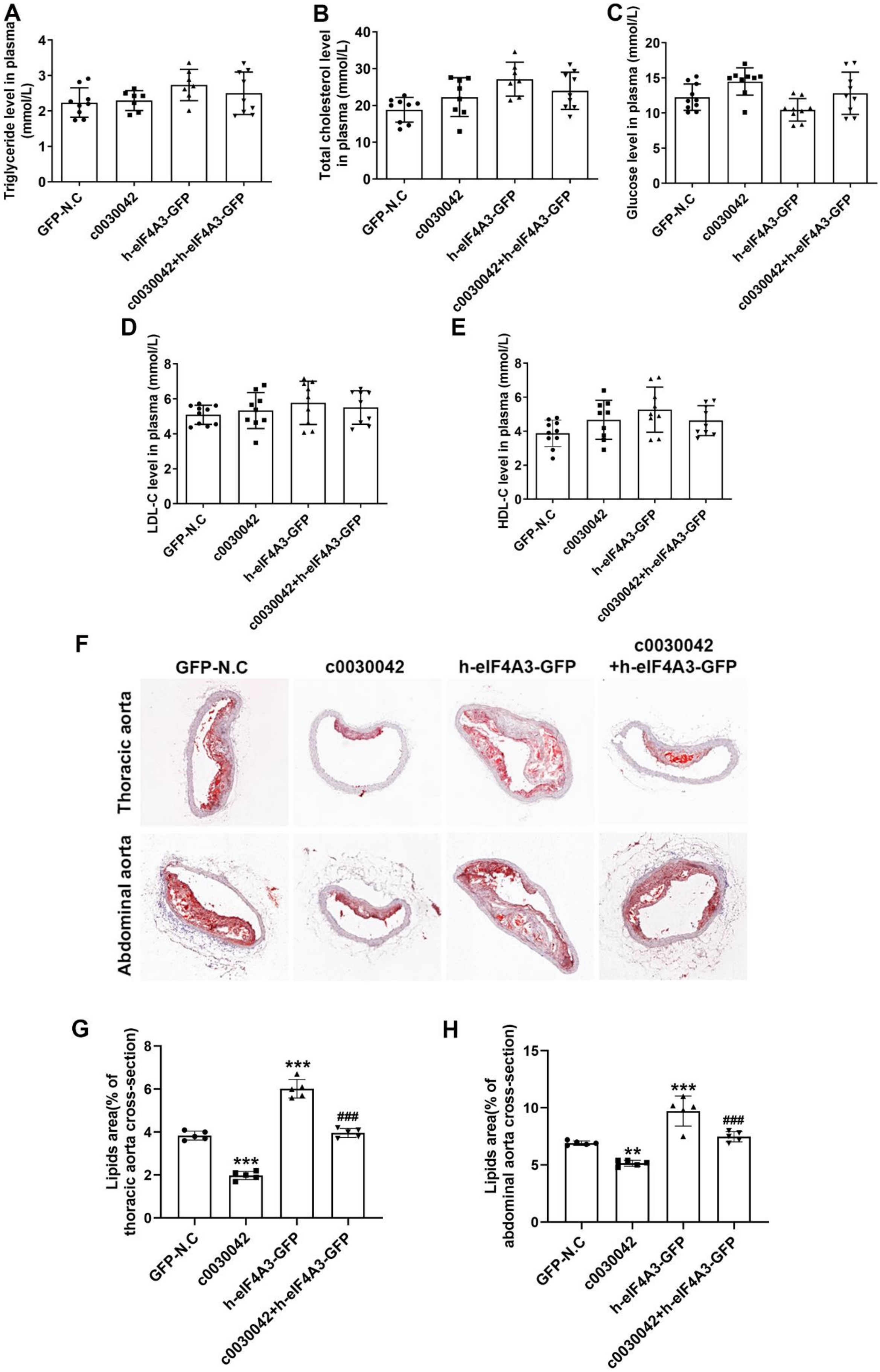

Supplementary Figure 5 The lipid level and deposition in four groups. **A - E** ELISA tested plasma level of triglyceride, total cholesterol, LDL-C, HDL-C and glucose in four groups of mice (GFP-N.C, c0030042, h-eIF4A3-GFP and c0030042+h-eIF4A3-GFP group). Data are presented as mean ± SD. One-way ANOVA. $n \geq 7$. **F – H** Oil Red O staining of cross section of thoracic and abdominal aortas and quantification in four groups of mice. Data are presented as mean ± SD. One-way ANOVA. Compared with GFP-N.C, $^{**}p < 0.01$, $^{***}p < 0.001$; compared with h-eIF4A3-GFP, $^{\#\#\#}p < 0.001$). $n = 6$.
